# Phase I/II clinical trial of nivolumab in combination with oligo-fractionated irradiation for unresectable advanced or recurrent gastric cancer

**DOI:** 10.1038/s43856-023-00343-4

**Published:** 2023-08-15

**Authors:** Kosaku Mimura, Takashi Ogata, Yuya Yoshimoto, Daisaku Yoshida, Shotaro Nakajima, Hisashi Sato, Nozomu Machida, Takanobu Yamada, Yohei Watanabe, Tomoaki Tamaki, Hirohito Fujikawa, Yasuhiro Inokuchi, Suguru Hayase, Hiroyuki Hanayama, Zenichiro Saze, Hiroyuki Katoh, Fumiaki Takahashi, Takashi Oshima, Yoshiyuki Suzuki, Koji Kono

**Affiliations:** 1 Department of Gastrointestinal Tract Surgery, Fukushima Medical University School of Medicine, 1 Hikarigaoka, Fukushima, 960-1295 Japan; 2 Department of Blood Transfusion and Transplantation Immunology, Fukushima Medical University School of Medicine, 1 Hikarigaoka, Fukushima, 960-1295 Japan; 3 Department of Gastrointestinal Surgery, Kanagawa Cancer Center, 2-3-2 Nakao Asahi, Yokohama, 241-8515 Japan; 4 Department of Radiation Oncology, Fukushima Medical University School of Medicine, 1 Hikarigaoka, Fukushima, 960-1295 Japan; 5 Department of Radiation Oncology, Kanagawa Cancer Center, 2-3-2 Nakao Asahi, Yokohama, 241-8515 Japan; 6 Department of Gastroenterology, Kanagawa Cancer Center, 2-3-2 Nakao Asahi, Yokohama, 241-8515 Japan; 7 Department of Information Science, Iwate Medical University, 1-1-1 Idaidori, Yahaba, Shiwa, Iwate 028-3694 Japan

**Keywords:** Cancer immunotherapy, Gastric cancer

## Abstract

**Background:**

Although immune checkpoint inhibitors (ICI) targeting for PD-1 axis is a promising approach for advanced gastric cancer (GC) patients, the response rate is still limited. Induction of synergistic effect of irradiation with ICI targeting for the PD-1 axis can be an attractive strategy. The aim of this study was to assess the effect of the combination of irradiation with anti-PD-1 therapy for advanced GC.

**Methods:**

We conducted a single-arm, phase I/II trial in GC patients treated with a combination of nivolumab and oligo-fractionated irradiation (22.5 Gy/5 fractions/5 days) (NCT03453164). Eligible patients (*n* = 40) had unresectable advanced or recurrent GC which progressed after primary and secondary chemotherapy with more than one lesion. The primary endpoint is the disease control rate (DCR) of non-irradiated target lesions and the secondary endpoints are the median survival time (MST), safety, and DCR of irradiated lesions.

**Results:**

We observe that the DCR for the non-irradiated target as the abscopal effect is 22.5% (90% confidence interval (CI), 12.3–36.0), and the DCR for the irradiated lesion is 40.0% (90% CI, 26.9–54.2). The median survival time is 230 days (95% CI, 157–330), and grade 3 and higher adverse events (AEs) are observed in 16 patients (39 %) with no obvious additional AEs when adding irradiation.

**Conclusions:**

The present study suggests that the combination of nivolumab with oligo-fractionated irradiation has the potential to induce a promising anti-tumor effect for advanced GC.

## Plain language summary

Immunotherapy is a type of treatment that triggers the immune system to kill cancers. Combining immunotherapy with radiotherapy may enhance its effects. We evaluated this in a clinical trial in which we treated patients with advanced or recurrent cancers of the stomach (gastric cancer) with a combination of immunotherapy and radiotherapy. The combination was able to control disease in a subset of patients and was safe, with no obvious additional adverse effects when adding radiotherapy. The median survival time—at which point half of the patients treated are still alive—was 230 days. While these results are promising, larger, more rigorous studies are needed to determine whether this combination therapy is better than alternative approaches to treating advanced or recurrent gastric cancers.

## Abstract (web summary)

Mimura et al. report findings from a single arm, phase I/II study of combined radiation and anti-PD-1 therapy in patients with advanced or recurrent gastric cancer. Combination therapy achieves a disease control rate of 22.5% for non-irradiated lesions via an abscopal effect, or 40% for irradiated lesions, and a median survival time of 230 days.

## Introduction

Gastric cancer (GC) is the second leading cause of cancer-related deaths and the sixth most frequent cancer worldwide (GLOBOCAN 2018)^1^. Inhibition of programmed cell death protein-1 (PD-1)/programmed death ligand-1 (PD-L1) axis with immune checkpoint inhibitors (ICI) including nivolumab and pembrolizumab has been emerging as a novel treatment strategy for advanced GC^2,3^. In the ATTRACTION-2 study, patients with unresectable advanced or recurrent GC treated with nivolumab as the 3rd line setting showed an objective response rate (ORR) of 11.2%^2^ and prolonged overall survival (OS). Furthermore, the CheckMate 649 study revealed that nivolumab plus chemotherapy resulted in significant improvements in OS and progression-free survival (PFS) versus chemotherapy alone, indicating that nivolumab plus chemotherapy represents a new standard first-line treatment for patients with advanced GC^4^. Although ICI is a promising approach for advanced GC patients, the response rate is still limited and thus developing novel strategies to maximize the efficacy of ICI is utmost necessary. Among one of them, the induction of synergistic effect of irradiation with ICI can be an attractive strategy.

It has been considered that radiotherapy is expected to induce immunogenic cell death (ICD), and the combination with ICI can result in enhanced anti-tumor immune response^5^. Irradiation induces tumor cell death and can elicit the release of novel immunogenic antigens which are taken up by dendritic cells and eventually result in the expansion of anti-tumor cytotoxic T lymphocytes (CTL), as reported by us and others^5,6^. However, the effectiveness of concurrent therapy of radiotherapy with ICI is not fully established yet, except in some clinical trials showing that irradiation followed by anti-cytotoxic T-lymphocyte-associated protein 4 (CTLA-4) antibody (Ab) treatment in lung cancer resulted in a promising synergistic clinical effect^7^, and that anti-PD-L1 therapy with chemo-radiation improved the PFS and OS in patients with non-small-cell lung cancer (NSCLC)^8,9^. Of note, mechanistic insights including immunological evaluation for ICD are largely unknown.

We conducted a single-arm, phase I/II trial in which we enrolled 41 advanced GC patients treated with the combination of nivolumab and oligo-fractionated irradiation (ClinicalTrials.gov, NCT03453164). Oligo-fractionated irradiation is defined as a short course (2–5 fractions) of hypo-fractionated radiation and it is reported to activate a specific immune response against the tumor in addition to direct cytotoxic effect^10–12^. The aim of this study was to assess the clinical effect of the combination of oligo-fractionated irradiation with ICI for advanced GC. Our findings show that the combination of nivolumab with oligo-fractionated irradiation has a promising clinical effect of MST of 230 days without obvious additional adverse events (AEs).

## Methods

### Eligibility criteria

Patients enrolled in this study had unresectable advanced or recurrent GC that was intolerance or had progressed after primary and secondary chemotherapy, with more than one lesion assessable in diagnostic imaging (one lesion must be ≥2 cm). The study protocol of this trial is provided with this paper. The trial was conducted in accordance with the ethical principles of the 1964 Declaration of Helsinki and its later amendments (ClinicalTrials.gov identifier: NCT03453164, Japan Registry of Clinical Trials identifier: jRCTs021180002, University Hospital Medical Information Network Clinical Trials Registry identifier: UMIN000031508). The trial protocol was approved by the Certified Review Board in Fukushima Medical University School of Medicine (Reference No. 18004) and all patients provided written informed consent before enrollment.

To be eligible to participate in this study, patients were required to meet the following criteria: (1) unresectable advance or recurrent GC with intolerance or progression after standard treatment (primary and secondary chemotherapy), (2) more than one measurable lesion defined by the Response Evaluation Criteria in Solid Tumors (RECIST) guideline version 1.1 in diagnostic imaging (whole-body contrast-enhanced CT or PET-CT) within 14 days before entry, with at least one lesion ≥2 cm, (3) age: 20≤, (4) eastern cooperative oncology group performance status (PS): 0–2, (5) no contraindication for nivolumab (anti-PD-1 Ab) administration, (6) no contraindication for radiotherapy, (7) the most recent laboratory results within 14 days before study entry fulfill the following: WBC ≥ 3000/μl, neutrophil ≥1500/μl, hemoglobin ≥9.0 g/dl, platelets ≥100,000/μl, total bilirubin ≤2.0 times the institutional standard upper limit (ISUL), AST (GOT) and ALT (GPT) ≤ 3.0 times ISUL (in case with liver metastasis, ≤5.0 times ISUL), serum creatinine ≤1.5 times ISUL or creatinine clearance ≥ 60 ml/min calculated with cockcroft-Gault equation, (8) expected survival ≥ 3 months.

Patients who met the following criteria were not eligible to enroll in this study: (1) no tumor lesions that can be irradiated, (2) metachronous and simultaneous overlapping cancers (excluding intraepithelial cancer of the uterine cervix, fully treated basal cell carcinoma of the skin, and malignant tumors that were treated more than 5 years ago and have not recurred), (3) a history of severe hypersensitivity reactions to other Ab products, (4) taking immunosuppressive drugs or corticosteroids (prednisone or prednisolone equivalent ≥ 15 mg/day), (5) active autoimmune diseases or a history of recurrent autoimmune diseases (patients with type-1 diabetes, hypothyroid controllable by hormone replacement therapy, and dermatosis without the need for systemic therapy are eligible), (6) complications or history of interstitial pneumonia or pulmonary fibrosis diagnosed by imaging studies or clinical findings, (7) presence of severe disease or medical conditions: severe nutritional deficiencies, transient ischemic attack within 180 days prior to enrollment, cerebral vascular attack within 180 days prior to enrollment, thrombus or thromboembolism within 180 days prior to enrollment, congestive heart failure (NYHA class III or IV), unstable angina, myocardial infarction within 12 months, severe arrhythmias requiring medication, conduction abnormalities such as AV block beyond the second degree, uncontrollable hypertension, liver cirrhosis (Child Class B or higher), mental disorders that may interfere with compliance with this study protocol, unstable diabetes, uncontrolled pericardial fluid, uncontrolled ascites, uncontrolled pleural effusions, diseases requiring anticoagulation therapy (excluding antiplatelet therapy including low-dose aspirin), and systemic infection with treatment, (8) pregnant or lactating female, (9) fertile female who are unwilling to use contraception, (10) fertile male who are not willing to use contraception during study drug administration and for 7 months after study completion (if the partners are fertile females), (11) prohibited previous treatment: within 56 days of registration; radioactive drugs (except radiopharmaceuticals for examination or diagnostic purposes), within 28 days of registration; corticosteroids (excluding temporary use and predonine or prednisolone equivalent ≤15 mg/day), immunosuppressant drugs, anti-cancer drugs, adhesive treatment of pleura or pericardium, surgery with general anesthesia, and unapproved drugs, within 14 days of registration; surgery with local or superficial anesthesia, (12) participating in other clinical trials or clinical studies (excludes those without intervention), (13) a positive HIV antigen/Ab test or HTLV-1 Ab test, (14) history of treatment using ONO-4538, anti-PD-1 Ab, anti-PD-L1 Ab, anti-PD-L2 Ab, anti-CD137 Ab, anti-CTLA-4 Ab, or other Ab or drug therapies for T-cell regulation, (15) determined by the investigator to be ineligible for participation in this study.

### Study procedure

This prospective, open-label trial was designed as a single-arm, phase I/II study at Fukushima Medical University Hospital and Kanagawa Cancer Center. Radiotherapy of a total 22.5 Gy/5 fractions/5 days was given to the largest or symptomatic lesion and the starting day of radiotherapy was set as day 1. Nivolumab was administered in the period of day 15–22 at a dose of 3 mg/kg or 240 mg/body and continued every 2 weeks to a total of 6 administrations.

### Enrollment and endpoints

The first patient was enrolled on March 28, 2018, the last patient was enrolled on July 02, 2020, and this study was completed on January 31, 2021. Based on a previous study of ATTRACTION-2^2^, the disease control rate (DCR) was set at 40%. At a one-sided significance level of 5% and a power of 80%, a sample size of 39 patients was needed to detect an additional 20% improvement expected from the study treatment and 41 patients (82.9 % males, 17.1 % females) were enrolled in this study. The primary endpoint for this trial is the DCR of non-irradiated target lesions and the secondary endpoints are the median survival time (MST), safety (grading and frequency of AEs), and DCR of irradiated lesions. All enrolled patients (*n* = 41) were subjected to the safety analysis. Since one patient whose target lesions did not meet the eligible criteria, 40 patients were included in the efficacy analysis including the DCR and MST.

Tumor responses were evaluated after 3- and 6-times administration of nivolumab, and then on day 120 and 180 or at the end of discontinuation according to RECIST guideline version 1.1. The DCR was defined as the total number of patients with complete response (CR)/partial response (PR)/stable disease (SD) divided by the number of eligible patients. The MST was defined as the time from the start date of radiotherapy until the date of death from any cause. Toxicities were graded based on the Common Terminology Criteria for Adverse Events (CTCAE) version 4.0. All AEs were summarized without regard to causal relationships to the study treatment and the worst toxicity grades based on CTCAE ver4.0 per subject were tabulated for AEs and toxicities in all enrolled patients. Overall survival and probability of survival rate are reported but were not pre-specified as endpoints in the Study Protocol.

### Statistical analysis

We used R software (version 4.0.3.) for statistical analyses in the present study. From the data regarding the response rates of the non-irradiated targets and irradiated lesions, the cumulative DCR and the Clopper & Pearson two-sided 90% confidence interval (CI) were determined. Data regarding AEs and toxicities were tabulated, and the Clopper & Pearson two-sided 95% CI was calculated. Kaplan–Meier estimates with log-rank tests were used to compare OS. Estimates of the MST and probability survival rate were calculated using the Kaplan-Meier method, along with a two-sided 95% CI for each using the Brookmeyer-Crowley method and Greenwood’s formula, respectively.

### Reporting summary

Further information on research design is available in the Nature Portfolio Reporting Summary linked to this article.

## Results

### Study protocol and patient characteristics

**Fig. 1:**
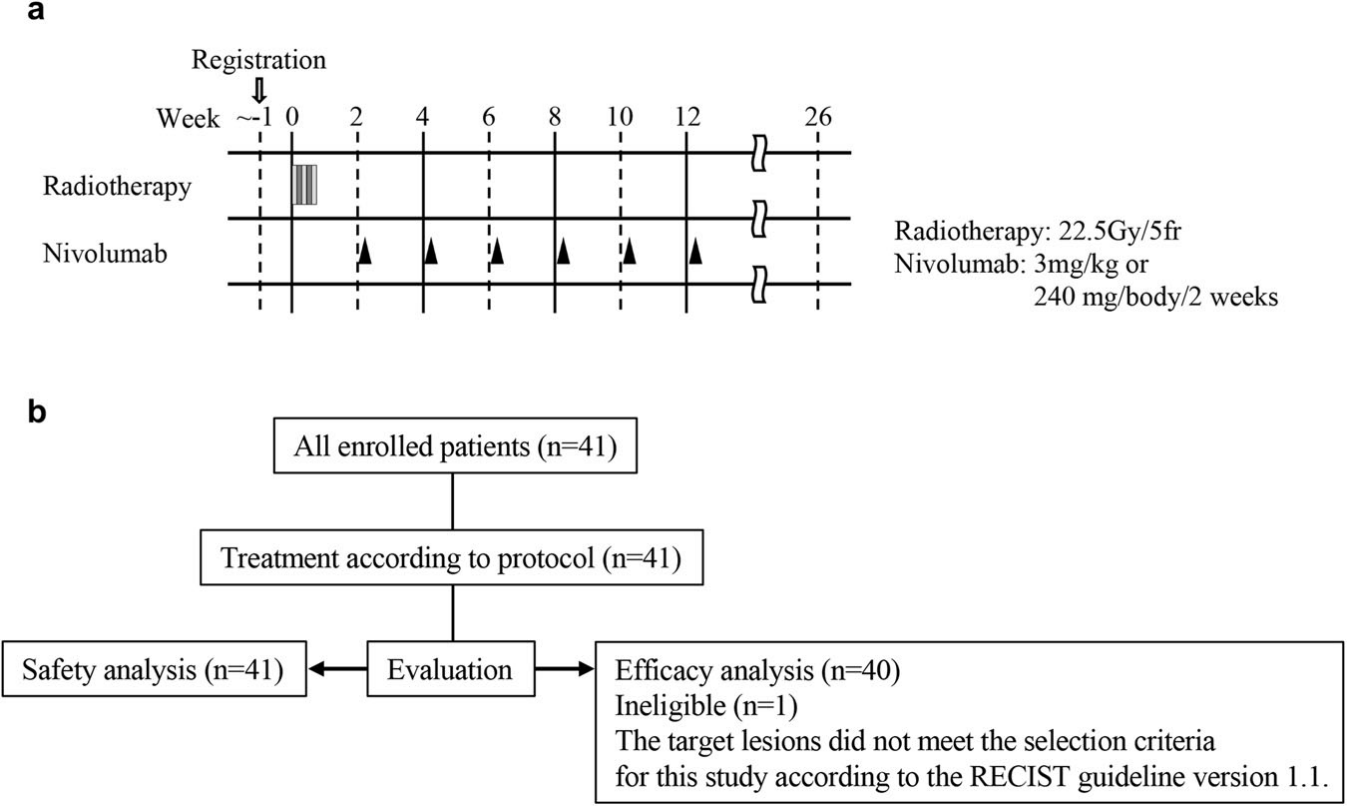
Study protocol and baseline characteristics. **a** Study protocol. **b** Eligibility for safety and efficacy analysis for all enrolled patients.

Unresectable advanced or recurrent GC patents (*n* = 41) who developed progression after primary and secondary chemotherapy, and matched to the inclusion criteria were enrolled and received the treatment according to the protocol (Fig. 1a). Safety analysis was performed in all 41 patients, while efficacy analysis was done in 40 subjects, since 1 patient was not eligible (Fig. 1b). As shown in Table 1, most of the patients had previously been heavily treated with chemotherapy and tumor burden was relatively high, since most of the patients had developed more than 5 measurable metastatic lesions with more than 3 organs involved.

**Table 1:** Characteristics of enrolled patients.

		Safety analysis		Efficacy analysis	
*n*		41		40	
Sex	Male	34	(82.9%)	33	(82.5%)
	Female	7	(17.1%)	7	(17.5%)
Age	Median (range)	70 (36–86)		70 (36–86)	
Prior chemotherapy	2 regimens	24	(58.5%)	23	(57.5%)
	3 regimens	17	(41.5%)	17	(42.5%)
ECOG PS	0	30	(73.2%)	30	(75.0%)
	1	8	(19.5%)	7	(17.5%)
	2	3	(7.3%)	3	(7.5%)
Number of cancer lesions	2	1	(2.4%)	1	(2.5%)
	3	7	(17.1%)	6	(15.0%)
	4	0	(0.0%)	0	(0.0%)
	5–9	15	(36.6%)	15	(37.5%)
	≧10	18	(43.9%)	18	(45.0%)
Number of organs with cancer	1	7	(17.1%)	7	(17.5%)
	2	11	(26.8%)	10	(25.0%)
	3	20	(48.8%)	20	(50.0%)
	4	2	(4.9%)	2	(5.0%)
	5	1	(2.4%)	1	(2.5%)

### Response rate of non-irradiated and irradiated lesions

**Fig. 2:**
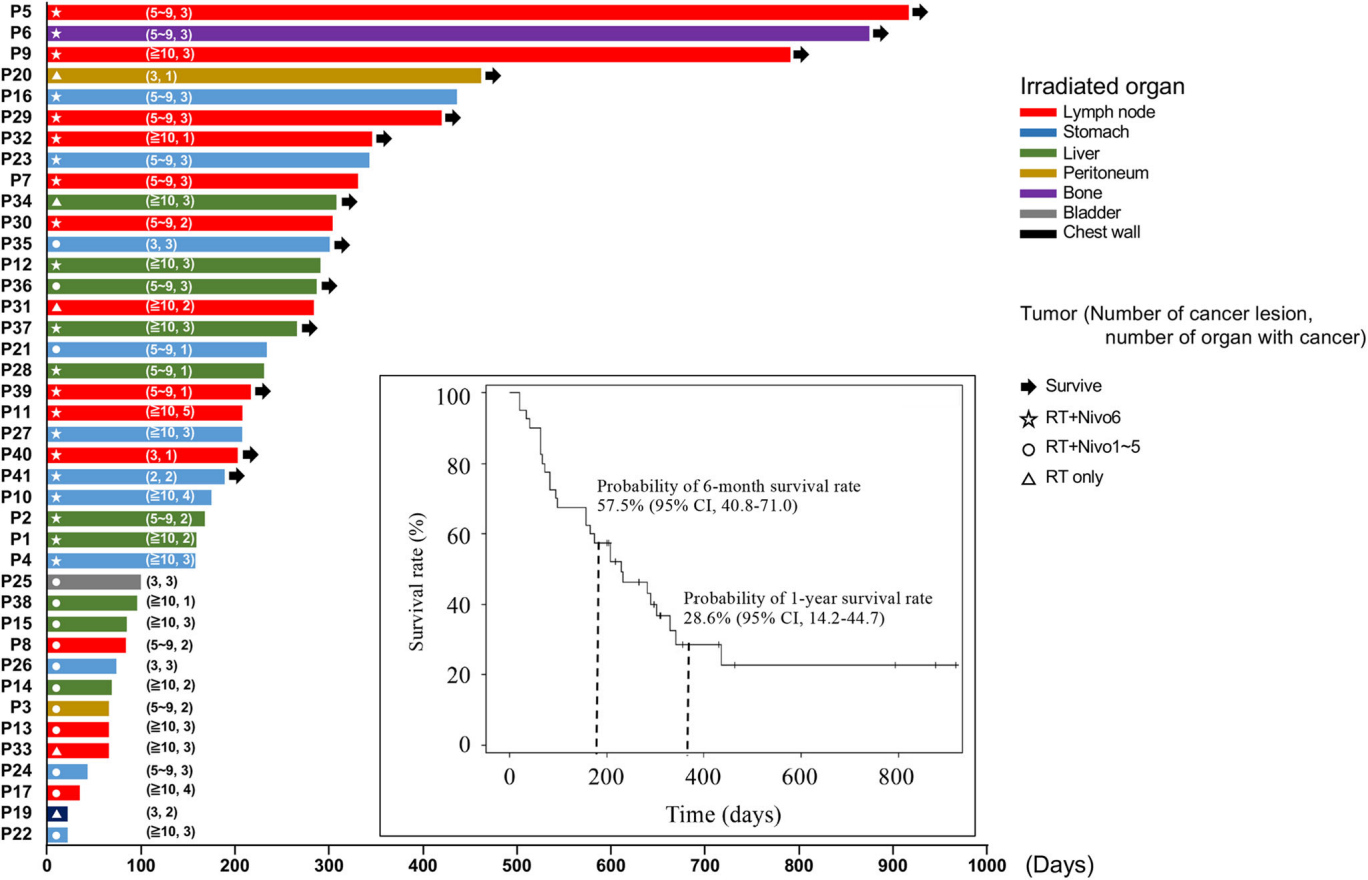
Clinical outcome in the enrolled patients. Swimmer plot: each lane represents a single patient’s data and *X*-axis represents the duration of the study protocol for each patient. P represents each patient’s identification number and each color of lane shows the irradiated organ. Survival rate in the dataset in efficacy analysis is shown (lower right). RT+Nivo6, radiotherapy + 6 administrations of nivolumab; RT+Nivo1–5, radiotherapy + 1–5 administrations of nivolumab; RT only, radiotherapy only.

The DCR (CR + PR + SD rate) for the non-irradiated target as abscopal effect was 22.5% (90% CI, 12.3–36.0) (primary endpoint), and the DCR for the irradiated lesion was 40.0% (90% CI, 26.9–54.2) (secondary endpoint) (Table 2). Furthermore, the DCR in non-irradiated and irradiated lesions in patients whose lymph nodes were irradiated were 35.7% (90% CI, 15.3–61.0) and 57.1% (90% CI, 32.5–79.4), respectively (Table 2). In the secondary endpoints, the MST was 230 days (95% CI, 157–330) (Fig. 2) and the safety profile showed that grade 3 and higher AEs were observed in 16 patients (39%) (Table 3), with no obvious additional AEs when adding irradiation.

**Table 2:** Response rate of non-irradiated targets and irradiated lesions.

		Non-irradiated lesions						Irradiated lesions					
		*N*	CR	PR	SD	PD/NE	% of CR/PR/SD (90%CI)	*N*	CR	PR	SD	PD/NE	% of CR/PR/SD (90%CI)
Total		40	1	5	3	31	22.5 (12.3–36.0)	40	5	6	5	24	40.0 (26.9–54.2)
Irradiated organ	Liver	10	1	0	0	9	10.0 (0.5–39.4)	10	1	0	2	7	30.0 (8.7–60.7)
	Peritoneum	2	0	0	0	2	0.0 (0.0–77.6)	2	0	0	0	2	0.0 (0.0–77.6)
	Stomach	11	0	2	1	8	27.3 (7.9–56.4)	11	0	3	1	7	36.4 (13.5–65.0)
	Lymph node	14	0	3	2	9	35.7 (15.3–61.0)	14	3	3	2	6	57.1 (32.5–79.4)
	Bone	1	0	0	0	1	0.0 (0.0–95.0)	1	1	0	0	0	100.0 (5.0–100.0)
	Chest wall	1	0	0	0	1	0.0 (0.0–95.0)	1	0	0	0	1	0.0 (0.0–95.0)
	Bladder	1	0	0	0	1	0.0 (0.0–95.0)	1	0	0	0	1	0.0 (0.0–95.0)
Study protocol	RT only	7	0	0	0	7	0.0 (0.0–34.8)	7	0	0	0	7	0.0 (0.0–34.8)
	RT+Nivo1	4	0	0	0	4	0.0 (0.0–52.7)	4	0	0	0	4	0.0 (0.0–52.7)
	RT+Nivo2	1	0	0	0	1	0.0 (0.0–95.0)	1	0	0	0	1	0.0 (0.0–95.0)
	RT+Nivo3	6	0	0	0	6	0.0 (0.0–39.3)	6	0	0	0	6	0.0 (0.0–39.3)
	RT+Nivo4	1	0	0	0	1	0.0 (0.0–95.0)	1	0	0	0	1	0.0 (0.0–95.0)
	RT+Nivo6	21	1	5	3	12	42.9 (24.5–62.8)	21	5	6	5	5	76.2 (56.3–90.1)

*N* total number, *CR* complete response, *PR* partial response, *SD* stable disease, *PD* progressive disease, *NE* inevaluavle for response, *RT* only, radiotherapy only, *RT+Nivo1* radiotherapy + 1 administration of nivolumab, *RT+Nivo2* radiotherapy + 2 administrations of nivolumab, *RT+Nivo* radiotherapy + 3 administrations of nivolumab, *RT+Nivo4* radiotherapy + 4 administrations of nivolumab, *RT+Nivo6* radiotherapy + 6 administrations of nivolumab.

**Table 3:** Frequency of AEs.

CTCAE ver.4.0	Gr.1	Gr.2	Gr.3	Gr.4	%Gr.3–4 (95%CI)
Abdominal pain	2	0	0	0	0.0 (0.0–8.6)
Alanine aminotransferase increased	5	0	0	0	0.0 (0.0–8.6)
Alkaline phosphatase increased	4	5	1	0	2.4 (0.0–12.8)
Anemia	5	6	6	2	19.5 (8.8–34.8)
Anorexia	4	4	5	0	12.1 (4.0–26.2)
Arthralgia	1	0	0	0	0.0 (0.0–8.6)
Ascites	0	2	0	0	0.0 (0.0–8.6)
Aspartate aminotransferase increased	7	1	0	0	0.0 (0.0–8.6)
Blood bilirubin increased	1	0	1	0	2.4 (0.0–12.8)
Cholecystitis	0	0	1	0	2.4 (0.0–12.8)
Constipation	3	2	0	0	0.0 (0.0–8.6)
Creatinine increased	3	1	0	0	0.0 (0.0–8.6)
Dehydration	0	0	2	0	4.8 (0.5–16.5)
Diarrhea	2	1	0	0	0.0 (0.0–8.6)
Dyspnea	0	1	1	0	2.4 (0.0–12.8)
Fatigue	8	1	0	0	0.0 (0.0–8.6)
Febrile neutropenia	0	0	1	0	2.4 (0.0–12.8)
Fever	4	0	1	0	2.4 (0.0–12.8)
Gastric hemorrhage	0	0	1	0	2.4 (0.0–12.8)
Gum infection	0	1	0	0	0.0 (0.0–8.6)
Headache	1	0	0	0	0.0 (0.0–8.6)
Hyperglycemia	8	7	1	0	2.4 (0.0–12.8)
Hyperkalemia	0	1	1	0	2.4 (0.0–12.8)
Hypertension	1	3	0	0	0.0 (0.0–8.6)
Hyperuricemia	2	0	1	0	2.4 (0.0–12.8)
Hypoalbuminemia	4	8	1	0	2.4 (0.0–12.8)
Hypocalcemia	4	4	0	0	0.0 (0.0–8.6)
Hypoglycemia	0	1	0	0	0.0 (0.0–8.6)
Hypokalemia	1	0	0	0	0.0 (0.0–8.6)
Hyponatremia	5	0	1	0	2.4 (0.0–12.8)
Hypothyroidism	0	2	0	0	0.0 (0.0–8.6)
Ileus	0	0	1	0	2.4 (0.0–12.8)
Malaise	0	2	0	0	0.0 (0.0–8.6)
Myalgia	1	0	0	0	0.0 (0.0–8.6)
Nausea	4	3	2	0	4.8 (0.5–16.5)
Neutrophil count decreased	1	1	0	0	0.0 (0.0–8.6)
Peripheral motor neuropathy	1	0	0	0	0.0 (0.0–8.6)
Peripheral sensory neuropathy	5	0	0	0	0.0 (0.0–8.6)
Platelet count decreased	2	1	1	0	2.4 (0.0–12.8)
Proteinuria	1	0	1	0	2.4 (0.0–12.8)
Pruritus	5	1	0	0	0.0 (0.0–8.6)
Rash maculo-papular	2	0	0	0	0.0 (0–8.6)
Small intestinal obstruction	0	1	0	0	0.0 (0.0–8.6)
Supraventricular tachycardia	0	0	0	1	2.4 (0.0–12.8)
Tumor pain	3	0	3	0	7.3 (1.5–19.9)
Vomiting	3	3	0	0	0.0 (0.0–8.6)
White blood cell decreased	1	3	1	0	2.4 (0.0–2.8)
Edema limbs	5	0	0	0	0.0 (0.0–8.6)
Lung infection	0	0	1	0	2.4 (0.0–12.8)
Generalized muscle weakness	1	0	0	0	0.0 (0.0–8.6)

CTCAE ver.4.0, Common Terminology Criteria for Adverse Events Version 4.0; Gr, grade.

### Survival (additional outcomes)

The swimmer plots presented in Fig. 2, 13 patients were still alive at the date of data fixation, and the OS curve, as shown in Fig. 2, probability of 1-year survival rate was 28.6% (95% CI, 14.2–44.7). There were no significant differences in the survival between modified Glasgow Prognostic Score (mGPS) A (*n* = 19) and mGPS B-D (*n* = 21) groups (*p* = 0.0572), between PS 0 (*n* = 30) and PS 1–2 groups (*n* = 10) (*p* = 0.0739) (Supplementary Fig. 1). In addition, there was also no significant difference in survival associated with irradiated organs among liver (*n* = 10), stomach (*n* = 11), lymph node (*n* = 14), and others (*n* = 5) groups (*p* = 0.6628), number of cancer lesions between 2–9 (*n* = 22) and ≥10 (*n* = 18) groups (*p* = 0.1163), white blood cell counts between above (*n* = 20) and below (*n* = 20) the median groups (*p* = 0.6083), lymphocyte counts between above (*n* = 20) and below (*n* = 20) the median groups (*p* = 0.3353), neutrophil-to-lymphocyte ratio between above (*n* = 20) and below (*n* = 20) the median groups (*p* = 0.5455), and grade of AEs between grade 1–2 (*n* = 24) and 3–4 groups (*n* = 16) (*p* = 0.1098).

## Discussion

The current trial resulted in a promising clinical effect, with MST of 230 days without obvious additional AEs, as compared to MST of 156 days in the ATTRACTION-2 study^2^. Because of the inclusion criteria in this clinical trial, patients had more than one measurable lesion in 3rd line setting, indicating that the tumor burden in this clinical trial cohort was more severe compared to what was observed in the ATTRACTION-2. Considering this background, the clinical efficacy of this combination strategy seems to be promising. A further clinical study with a pivotal RCT would be needed to draw impactful conclusions on the clinical benefit of the combination of nivolumab with radiation.

The combined immunotherapy utilizing ICI targeting for the PD-1 axis and irradiation is an attractive treatment strategy. In fact, anti-PD-L1 therapy after chemo-radiation improved the PFS and OS in patients with NSCLC (PACIFIC trial)^8,9^, and induction treatment with irradiation followed by nivolumab provided clinical benefit in patients with metastatic triple-negative breast cancer (TONIC trial)^13^. Moreover, it was recently reported that neoadjuvant therapy using anti-PD-1/PD-L1 therapy and irradiation is associated with a favorable pathological response such as pathological CR and PR in patients with locally advanced rectal cancer, locally advanced head and neck squamous cell carcinoma, and early-stage NSCLC^14–16^. These reports suggest that combined immunotherapy utilizing nivolumab and irradiation may well be effective in patients with GC. Therefore, we conducted a single-arm, phase I/II trial of nivolumab in combination with oligo-fractionated irradiation for unresectable advanced or recurrent GC patients to evaluate the efficacy and safety of this combination therapy.

With respect to the optimization of radiation-induced immunogenicity, it has been reported that activation of c-GAS/STING axis and its related chemokine profile is strongly associated with clinical response to the combination of radiation with ICI^17^. For example, a comparison of oligo-fractionated irradiation with a single shot of irradiation showed a completely different profile for c-GAS/STING activation and resulted in downstream recruitment of dendritic cells and activation of CD8(+) T cells^17^. It was also reported that oligo-fractionated irradiation was more effective in abscopal effect than the single, ablative dose of irradiation^12,18^. We have recently reported that irradiation can induce remodeling of the tumor-microenvironment through tumor cell-intrinsic expression of cGAS-STING^19^. Based on these translational data, for the current clinical trial, we designed the irradiation protocol with an oligo-fractionated schedule (22.5 Gy/5 fractions).

Moreover, it was recently reported that non-ablative oligo-fractionated irradiation may induce the abscopal response in murine model and patients with NSCLC^7,11^. On the other hand, pembrolizumab concomitant with irradiation (69.96 Gy/33 fractions) did not improve PFS and OS compared to cetuximab plus irradiation in patients with locally advanced squamous cell carcinoma of head and neck^20^, and addition of stereotactic body radiotherapy (24 Gy/3 fractions) did not improve efficacy of combined nivolumab and ipilimumab in patients with advanced Merkel cell carcinoma^21^. Furthermore, low-dose (2 Gy/4 fractions/2 days in the first four cycles of therapy) or hypo-fractionated (24 Gy/3 fractions in the first cycle only) radiotherapy did not increase overall response rates of PD-L1 plus CTLA-4 therapy in patients with NSCLC resistant to immunotherapy targeting for PD-1 axis^22^. Further clinical studies would be definitely needed to identify the optimal radiation condition in order to achieve better synergistic anti-tumor immunity induced by the combination of radiotherapy with ICI^23^. In addition, we revealed in this study that the DCRs in non-irradiated and irradiated lesions in patients whose lymph nodes were irradiated (35.7% and 57.1%) were higher than those in all enrolled patients (22.5% and 40.0%) (Table 2). Therefore, an oligo-fractionated schedule (22.5 Gy/5 fractions) may be an appropriate radiation condition for lymph nodes metastasis in advanced GC and we intend to also plan an improved version of this study that focuses on irradiation of the lymph node metastasis.

Taken together, the present study suggests that the combination of nivolumab with oligo-fractionated irradiation has a potential to induce a promising anti-tumor effect for advanced GC. Further clinical studies will be needed to draw solid conclusions on the clinical efficacy of this combination therapy.

### Supplementary information

Supplementary Data 1

Supplementary Information

Description of Additional Supplementary Files

Reporting Summary

## Data Availability

All relevant data supporting the findings of this study are included in this published article and its Supplementary information. Individual deidentified participant data to generate the findings of this study are available in Supplementary Data 1. The Study Protocol is provided within the Supplementary information.

## Code availability

All statistical analyses in the present study were performed using R software (version 4.0.3.). Further information on code used in the present study is obtained from the corresponding author on reasonable request.
