# Supplementary material for: Phase I/II clinical trial of nivolumab in combination with oligo-fractionated irradiation for unresectable advanced or recurrent gastric cancer
Source: Commun Med (Lond). 2023 Aug 15;3:111. doi: 10.1038/s43856-023-00343-4 (PMC10427681; doi:10.1038/s43856-023-00343-4)
Supplement: Supplementary file 2 — Supplementary Information [file 43856_2023_343_MOESM2_ESM.pdf]

Phase I/II clinical trial of nivolumab in combination with oligo-fractionated irradiation for unresectable advanced or recurrent gastric cancer

Kosaku Mimura[1, 2], Takashi Ogata[3], Yuya Yoshimoto[4], Daisaku Yoshida[5], Shotaro Nakajima[1], Hisashi Sato[4], Nozomu Machida[6], Takanobu Yamada[3], Yohei Watanabe[1], Tomoaki Tamaki[4], Hirohito Fujikawa[3], Yasuhiro Inokuchi[6], Suguru Hayase[1], Hiroyuki Hanayama[1], Zenichiro Saze[1], Hiroyuki Katoh[5], Fumiaki Takahashi[7], Takashi Oshima[3], Yoshiyuki Suzuki[4], and Koji Kono[1]

1) Department of Gastrointestinal Tract Surgery, Fukushima Medical University School of Medicine, 1 Hikarigaoka, Fukushima, 960-1295, Japan
2) Department of Blood Transfusion and Transplantation Immunology, Fukushima Medical University School of Medicine, 1 Hikarigaoka, Fukushima, 960-1295, Japan
3) Department of Gastrointestinal Surgery, Kanagawa Cancer Center, 2-3-2 Nakao Asahi, Yokohama, 241-8515, Japan
4)_Department of Radiation Oncology, Fukushima Medical University School of Medicine, 1 Hikarigaoka, Fukushima, 960-1295, Japan
5) Department of Radiation Oncology, Kanagawa Cancer Center, 2-3-2 Nakao Asahi, Yokohama, 241-8515, Japan
6) Department of Gastroenterology, Kanagawa Cancer Center, 2-3-2 Nakao Asahi, Yokohama, 241-8515, Japan
7) Department of Information Science, Iwate Medical University, 1-1-1 Idaidori, Yahaba, Shiwa, Iwate, 028-3694, Japan

To whom correspondence should be addressed: kojikono@fmu.ac.jp
ORCID: https://orcid.org/0000-0003-0106-2767

Supplementary Information

Supplementary Figure

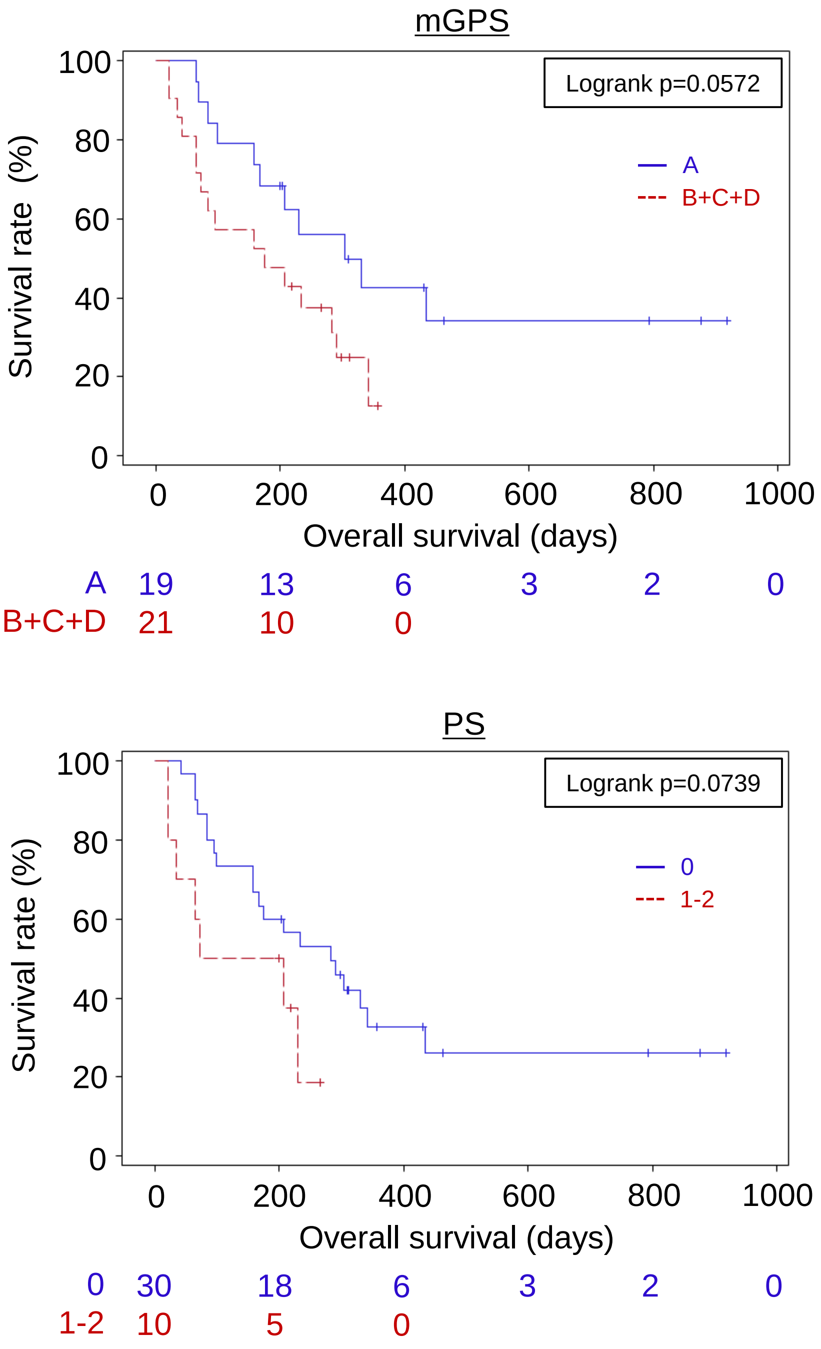

Supplementary Figure 1. Probability of survival for patients in the study protocol, modified Glasgow Prognostic Score (mGPS), and performance status (PS). Survival rate was compared between the groups that mGPS A (n=19) with mGPS B-D (n=21) and PS 0 (n=30) with PS 1-2 (n=10).

Phase I/II Clinical Trial of Nivolumab (Anti-PD-1 Antibody) in Combination with Local Radiation Therapy for Unresectable Advanced or Recurrent Gastric Cancer Refractory to Standard Therapy

CIRCUIT trial

Principal Investigator: Koji Kono

Gastrointestinal Tract Surgery, Fukushima Medical University Hospital
1 Hikarigaoka, Fukushima-city, Fukushima 960-1295
TEL: 024-547-1111 (extension 2342)
E-mail: kojikono@fmu.ac.jp

Research Plan Summary

Purpose of Research

Patients with unresectable advanced or recurrent gastric cancer with intolerance or progression for standard treatment (primary and secondary chemotherapy) will receive short-term radiation therapy on selected lesions prior to of nivolumab (anti-PD-1 antibody) therapy, which is approved as a third-line chemotherapy, to determine its safety and efficacy (enhancement of the systemic effect of nivolumab and sensitization of the local effect of radiation).

Method of Research

Candidates: Patients with unresectable advanced or recurrent gastric cancer who are diagnosed as ineffective (intolerant or progression) to standard treatment (first-line or second-line chemotherapy) and have two or more lesions that can be confirmed by imaging.

↓

Preliminary review, confirmation of eligibility criteria, acquisition of consent to participate in this study, registration

↓

Treatment = Radiotherapy + Nivolumab (anti-PD-1 mAb)

① **Radiotherapy**: If the patient has symptoms such as pain or paralysis, irradiate the lesion thought to be the main cause of symptoms, or if asymptomatic, irradiate the largest lesion (or the second largest lesion if radiotherapy to the largest lesion is difficult) with 4.5 Gy once a day for 5 days (total 22.5 Gy/5 times/5 days). Day 1 is the start date of radiotherapy.

② **Nivolumab**: Start administration between Days 15–22. Nivolumab is administered at 3 mg/kg body weight or 240 mg every 2 weeks for a total of six doses.

↓

Observation and Evaluation of Efficacy

Evaluation Items

Primary endpoint: 1) Disease control rate

Secondary endpoints: 1) Median survival time,

2) Safety (grade and frequency of adverse events),

3) Local control rate

4) Immunological parameters

・**Expected Number of Cases**

40 cases

Duration of Study

Registration period: March 1, 2018 to August 31, 2020 (2 years and 6 months)

Total research period: March 1, 2018 to August 31, 2021 (3 years and 6 months)

Research Subject Selection Policy

Patients with unresectable advanced or recurrent gastric cancer who are diagnosed as ineffective (intolerant or progression) to standard treatment (first-line or second-line chemotherapy) and have two or more lesions that can be confirmed by imaging.

Table of Contents

1. Purpose and significance of research ... 6
1.1. Background and Significance ... 6
1.2. Objectives of the study ... 7
2. Methods and duration of the study ... 7
2.1. Schema ... 7
2.2. Research design ... 8
2.3. Treatment and intervention plans ... 8
2.4. Combination therapy ... 11
2.5. Criteria for changes in dosage/use ... 12
2.6. Evaluation items ... 13
2.7. Observation and examination items ... 15
2.8. Expected number of cases ... 20
2.9. Case registration ... 20
2.10. Statistical analysis ... 21
2.11. During the study ... 22
3. Policy for selecting research subjects ... 22
3.1. Inclusion criteria ... 22
3.2. Exclusion criteria ... 23
4. The scientific rationale for the research ... 24
5. The procedure for obtaining informed consent ... 24
5.1. Explanation and consent of research subjects ... 24
5.2. Procedures for obtaining informed consent from a substitute or other person ... 25
5.3. Procedures for obtaining informed assent ... 25
6. Handling of Adverse Events ... 25
6.1. Response to Adverse Events ... 25
6.2. Response to the occurrence of serious adverse events ... 25
7. Criteria for discontinuation and completion ... 26
8. Data management methods ... 27
8.1. Prepare and filling out a Case Report Form (CRF) ... 27
8.2. Sending and storing Case Report Form (CRF) ... 27
8.3. Procedures for amending CRF ... 27
8.4. Identification of source material ... 28
9. Transfer of samples and information to and from other institution ... 28
10. Method of storage and disposal of samples and information ... 28
10.1. Storage ... 28

10.2. Disposal 29
11. Handling of personal data 30
11.1. Method of use 30
11.2. Security management responsibility system 30
12. The burdens and foreseeable risks and benefits to the research subjects, the comprehensive assessment of them and measures to minimize such burdens and risks 30
13. The status of funding sources for research and the conflicts of interest related to research of the research institution, individual revenues, and attending doctors, etc. 31
14. Methods of disclosing information on research 32
14.1. Registration of the research plan 32
14.2. Publication of research results 33
15. Report 34
16. Response to consultation from research subjects and others concerned 34
17. Handling of research in situations where there is an immediate and obvious life-threatening situation for the research subject 34
18. In cases involving financial burdens or gratuities to research subjects 34
19. In the case of research involving invasive procedures, any compensation for damage to health caused by the research 35
20. In the case of research involving medical treatment beyond normal medical treatment, handling of provision of medical treatment to research subjects after completion of the research 35
21. Handling of research results (including incidental findings) in relation to research subjects for cases in which the conduct of research may lead to important findings, such as the health of the research subjects, genetic characteristics that can be passed on to their offspring, etc. 35
22. Possibility that the sample/information will be used for future research not identified at the time when consent is received 35
23. Quality control implementation system and procedures for clinical trials 35
23.1. Monitoring 36
23.2. Data center 36
23.3. Biostatistics 36
23.4. Audits 36
24. Research implementation organization 36
25. Outsourced research and assay 37
26. Citations 38
27. Appendix 38

1. Purpose and significance of research

1.1. Background and Significance

Treatment of unresectable advanced or recurrent gastric cancer

Chemotherapy is the first line treatment option that is considered for unresectable advanced or recurrent gastric cancer. Currently, in Japan, the combination therapy of oral fluoropyrimidine antineoplastic agents (S-1, capecitabine) plus platinum (cisplatin, oxaliplatin) is recommended as first-line treatment for HER2-negative gastric cancer, and ramucirumab plus paclitaxel as second-line treatment. Trastuzumab combination therapy is also recommended for HER2-positive gastric cancer (1). Recent advances in chemotherapy have allowed a high degree of tumor reduction in unresectable advanced or recurrent gastric cancer, but the median survival time is approximately 6–13 months based on the results of national and international clinical trials, and the prognosis is still poor.

In 2017, the results of a multicenter international clinical trial of the immune checkpoint inhibitor, nivolumab (anti-PD-1 antibody (Ab)), in unresectable advanced or recurrent gastric cancer that was refractory or intolerant to standard treatment were reported, showing a significant overall survival (OS) benefit (hazard ratio [HR]: 0.63; 95% confidence interval. [CI]: 0.50–0.78; $p < 0.0001$) and it was approved as a tertiary chemotherapy in Japan in 2017. However, even in the aforementioned report, the response rate was not as high as expected at 11.2% (95% confidence interval: 7.7–15.6), and the development of new treatments and combination therapies is urgently needed.

Radiotherapy is used for various malignant tumors throughout the body, but for advanced or recurrent gastric cancer that is not curatively resectable, especially in cases with multiple foci, treatment is only performed in symptomatic and conservative approaches based on the tolerable dose of the surrounding tissues and the therapeutic effect (radiosensitivity).

Combination therapy with radiotherapy and immune checkpoint inhibitors

Although there is no established consensus on the efficacy (additive or synergistic effect) of the combination of radiotherapy and immunotherapy, its efficacy has been reported in many basic studies, translational studies, case reports, and relatively small clinical studies in recent years. We have also reported the induction of cancer antigen-specific T lymphocytes after irradiation in patients with esophageal squamous cell carcinoma (2) and the drug-resistant/unresectable recurrent gastric cancer showing an abscopal effect in combination with immuno-cell therapy (3). The mechanism is thought that irradiation-induced cell death of cancer cells became new antigen and a tumor immune response was activated in vivo.

Significance of this examination

This clinical trial is designed to evaluate the additive effect to nivolumab and radiotherapy in patients

with unresectable advanced or recurrent gastric cancer who have been diagnosed to have an inadequate response (intolerance or progression: PD) to standard (first- and second-line) chemotherapy by combining radiation therapy immediately before the third-line nivolumab treatment. The trial will be positioned as a proof of concept (POC) to explore the efficacy of this therapy by conducting exploratory clinical and immunological analyses as a phase I/II study. The safety of this combination therapy will also be investigated as a secondary study. It is positioned to provide basic data on median survival time (MST) and percentage of local control for future randomized controlled trials (RCTs) of this therapy.

1.2. Objectives of the study

Eligible patients will be those with unresectable advanced or recurrent gastric cancer who have been diagnosed as having an inadequate respond (intolerance or progression: PD) to standard therapy (primary or secondary chemotherapy) and who have at least two image-confirmable lesions (one of which is at least 2 cm in length). To evaluate the safety and efficacy of the combination immunotherapy by administering short-term radiotherapy on selected lesions prior to nivolumab (anti-PD-1 Ab) treatment, which is approved as a third-line chemotherapy, in eligible patients, the following items will be assessed:

① Primary endpoint: disease control rate

② Secondary endpoint:

1. Median survival time (MST)
2. Safety (grade and frequency of adverse events)
3. Local control rate
4. Immunological parameters:

   Histological examination

   ・Analysis of the tumor microenvironment by immunostaining.

   Peripheral blood test

   ・Analysis of peripheral blood lymphocyte subset

   ・Measurement of cytokines in plasma

   ・Analysis of regulatory T cell function

   ・Analysis of antigen-specific T cell function

2. Methods and duration of the study

2.1. Schema

・Patients with unresectable advanced or recurrent gastric cancer.
・Patients who are diagnosed as ineffective (intolerant or progression) to standard treatment (first-line or second-line chemotherapy) and have two or more lesions that can be confirmed by imaging.
・40 cases

↓

Obtaining consent to participate in the trial, checking eligibility criteria, enrolment

↓

Treatment = radiotherapy ≫ Nivolumab (anti-PD-1 Ab)
Radiotherapy: 4.5 Gy once daily for 5 days (total 22.5 Gy/5 times/5 days) to the lesion thought to be the main cause of symptoms if there are symptoms such as pain or paralysis, or to the largest lesion if asymptomatic (second largest lesion if radiotherapy to the largest lesion is difficult). The day that radiotherapy is started is Day 1.
Nivolumab: Dosing is started between Days 15 and 22; Nivolumab is administered at 3 mg/kg (body weight) or 240 mg every 2 weeks for a total of six doses.

End of administration

↓

Follow-up (〜Day 180 ± 14) and efficacy assessment (Day 180 ± 14)

End of trial

2.2. Research design

Phase I/II, single-arm, open (non-randomized), prospective study

2.3. Treatment and intervention plans

(1) Test chemicals/test equipment

Radiation therapy X-rays or electron beams generated by radiation therapy equipment installed in the Department of Radiation Therapy at Fukushima Medical University

Hospital and the Department of Radiation Oncology at Kanagawa Cancer Center.

Nivolumab (Anti-PD-1 Ab) Ono Pharmaceutical Industries Ltd/Bristol-Myers Squibb Co.
Opdivo intravenous infusion 20 mg, 100mg

(2) Dosage/applications

Radiotherapy

Exposure 22.5 Gy/5 times/5 days

Irradiation range

In accordance with the usual treatment methods used in symptomatic and palliative treatment, only the tumor is targeted and the irradiated area has a margin that takes into account the physical uncertainties assumed at each irradiated site (i.e. the clinical and prophylactic irradiation area is not considered).

Basis for setting

Prognosis and early initiation of nivolumab therapy are priorities, and the main target lesions were assumed to be liver metastases, lymph node metastases, local (surgical) recurrent lesions and bone metastases. Therefore, the radiotherapy period was kept short and the irradiation dose was set at 22.5 Gy/5 times/5 days, which is below the tolerable dose at each site and close to the tolerable dose in the brain and spinal cord.

Selection of irradiated tumors

If symptoms such as pain or paralysis are present, the lesion thought to be the main cause of symptoms should be selected; if asymptomatic, the largest lesion (or the second largest if radiotherapy of the largest lesion is difficult) should be selected. Tumors larger than 2 cm should be selected, taking into account the possibility that tumors that are too small may not cause an adequate biological response (induction of anti-tumor immunity).

Irradiation day interval

Radiotherapy is performed on consecutive Department of Radiation Therapy working days (weekdays and consecutive days). This does not apply during the end of the year and New Year holidays, national holidays and other holidays.). The day that radiotherapy is started is Day 1. If radiotherapy is not completed by Day 14, the patient is considered to have dropped out.

Irradiation implementation conditions

On each irradiation day, ensure that all of the following conditions are met before the day's irradiation

1） Body temperature: < 38.5°C (axilla)
2） Confirmation by the attending doctor that there are no problems with radiotherapy.

If the above criteria are not met, irradiation is postponed on a day-to-day basis until the criteria are met.

Nivolumab (anti-PD-1 Ab) treatment

Dosage and administration

In accordance with the recommended treatment for unresectable advanced or recurrent gastric cancer, 3 mg/kg (body weight) of nivolumab once every two weeks over at least one hour, or 240 mg once every two weeks over at least 30 minutes, is administered via intravenous infusion.

A total of six administered doses is considered the completion of protocol treatment, but there are no specific provisions for treatment after completion.

Continuation of treatment with nivolumab is also permitted within the insurance coverage at the discretion of the attending doctor.

However, any 'unacceptable concomitant or supportive therapy' as defined in 2.4.3) shall be considered a protocol deviation.

Dosing conditions

All of the following conditions should be met when starting administration of this drug.

1) White blood cell count ≥ 3,000/μL and neutrophil count ≥ 1,500/μL

2) Hemoglobin ≥ 9.0 g/dL

3) Platelets ≥ 100,000/μL

4) Total bilirubin ≤ 2.0 times the upper limit of the institutional reference value

5) AST (GOT) and ALT (GPT) ≤ 3.0 times the upper limit of the facility reference value. (If liver metastases are present, ≤ 5.0 times the upper limit of the institutional reference value.)

6) Serum creatinine ≤ 1.5 times the upper limit of the facility reference value or creatinine - clearance(Ccr) estimate ≥ 60 mL/min.

Basis for setting

It is expected that radiotherapy will enhance the efficacy of anti-tumor immunity. We speculate that the use of nivolumab in combination with post-irradiation therapy may induce an abscopal effect in some cases. We decided to start nivolumab between Days 15-22, with Day 1 as the start date of radiotherapy. Because IFN-γ and TNF-α in serum increased about 10-14 days after irradiation (mouse model) (4), and immune responses such as CTL activation were observed about 2 weeks after administration of peptide vaccines and activated dendritic cells (clinical observation).

A possible disadvantage of this treatment strategy is that the combination of radiotherapy and nivolumab may increase or intensify adverse events; however, as far as we have searched, there have been no reports of serious adverse events occurring.

2.4. Combination therapy

1) Acceptable concomitant and supportive therapies

The following concomitant and supportive care may be given as necessary. However, the name of the applicable event, the name of the drug used, its dosage, and the duration of use, etc., should be noted in the case report form. The drugs listed in this section are examples and do not limit the drugs used.

① Symptomatic treatment of Grade 1 or less interstitial lung disease and eye disorders caused by nivolumab or that of Grade 2 or less bronchospasm, diarrhea, colitis, neurotoxicity, hypersensitivity reactions, injection reactions (fever, chills, nausea, pain, headache, cough, scratchiness, rash, etc.) and thrombocytopenia. The dosage and administration of each drug used is in accordance with the package insert (insurance indications).

② Hepatoprotective drugs for the following liver function abnormalities caused by nivolumab. AST or ALT < 5–10 times the upper limit of the facility reference value for more than 2 weeks. AST or ALT < 10 times the upper limit of the facility reference value. Total bilirubin level < five times the upper limit of the facility reference value. Concomitant AST or ALT < three times the upper limit of the facility reference value and total bilirubin < two times the upper limit of the facility reference value. The dosage and administration of the respective drug should be in accordance with the accompanying documentation (insurance coverage).

③ Nausea, vomiting and anorexia (hostility) caused by radiotherapy. Metoclopramide (primperan) is tolerated to prevent symptom exacerbation. The standard of care for use is nausea, vomiting or anorexia of Grade 2 or above. The dosage and administration of metoclopramide (primperan) to be used should be in accordance with the package insert (insurance indication).

④ Radiotherapy-induced dermatitis.

The use of skin protectants such as hydride for Grade 1 dermatitis and topical corticosteroids for Grade 2 or higher skin rashes is acceptable. The dosage and administration of each drug should be in accordance with the package insert (insurance coverage).

⑤ Radiotherapy to other metastatic lesions.

During the course of nivolumab treatment, if palliative radiotherapy becomes necessary due to symptoms such as pain and bleeding worsen caused by an increase in lesions other than those treated with radiotherapy, radiotherapy should be allowed and observation continued if there are other evaluable lesions with no history of radiotherapy.

2) Unacceptable concomitant and supportive therapies

During protocol treatment (from the start of radiotherapy until after the sixth dose of nivolumab), none of the following treatments should be administered. Any of the following treatments are considered a protocol deviation.

① Treatment for cancers other than this protocol (chemotherapy, surgery, radiotherapy, endocrine therapy, immunotherapy, etc.). However, radiotherapy is permitted if the provisions of 2.4.1.⑤ are met.

② Other treatments presumed to influence the assessment of efficacy and safety in the study.

③ Immunosuppressive therapy. However, corticosteroids are only allowed in doses of hydrocortisone ≤ 200 mg/day for transfusion allergy and small-dose steroids (< 15 mg/day of prednisone) for the prevention of cerebral hypertension symptoms and adrenal insufficiency.

④ Administration of other study medicines, including development products.

2.5. Criteria for changes in dosage/use

1) Radiation dose escalation and dose reductions will not be performed. Nivolumab dosage and administration should be in accordance with the recommendations for the treatment of unresectable advanced or recurrent gastric cancer (Appendix).

2) Criteria for discontinuation of treatment

① Nivolumab (anti-PD-1 Ab) discontinuation criteria:

1) Failure to meet the criteria for initiation of nivolumab (anti-PD-1 Ab) in '2.3.(2) Nivolumab (anti-PD-1 Ab) treatment' and failure to resume treatment within 14 days of the planned treatment date.
2) If a patient requests discontinuation of nivolumab treatment, irrespective of whether or not it is associated with an adverse event.
3) Death during protocol treatment.
4) Ineligibility due to a known protocol violation, change of pathology diagnosis after enrolment, etc.
5) It is deemed inappropriate to continue treatment due to apparent worsening of clinical symptoms caused by disease progression.
6) Occurrence of Grade 2 or higher interstitial lung disease.
7) Occurrence of Grade 2 or higher eye disorders.
8) Occurrence of Grade 3 or higher bronchospasm, diarrhea, colitis, neurotoxicity, hypersensitivity reactions and injection reactions.
9) Occurrence of Grade 3 or higher thrombocytopenia.
10) Abnormalities in liver function that fulfill one of the following criteria have emerged. AST or ALT > 5–10 times the upper limit of the facility reference value for more than 2 weeks.

AST or ALT > 10 times the upper limit of the facility reference value. Total bilirubin levels > 5 times the upper limit of the institutional reference value. Concurrent AST or ALT > 3 times the upper limit of the facility reference value and total bilirubin > 2 times the upper limit of the facility reference value.

11) The principal investigator or a member of research team decides that continued treatment with nivolumab is not appropriate.

② Criteria for discontinuation of radiotherapy

Discontinue radiotherapy if any of the following occur:

1) Failure to meet the treatment criteria for radiotherapy in '2.3(2) – Radiotherapy'.
2) When the attending doctor decides that discontinuation is necessary due to an adverse event.
3) If the patient requests discontinuation of radiotherapy, irrespective of whether or not it is associated with an adverse event.
4) Patient deaths.
5) Ineligibility due to a known protocol violation, change of pathology diagnosis after enrolment, etc.
6) The principal investigator or a member of research team decides that continuation of treatment is not appropriate, taking into account efficacy or safety.

If this protocol treatment is discontinued, follow the decision of the attending doctor with regard to subsequent treatment. The reasons for discontinuation should be documented in the medical record and the case report form. If consent is withdrawn after treatment has started, it should be made as clear as possible whether the withdrawal of consent was due to ineffectiveness of the treatment or to an adverse or incidental event, and this should be record in order to assist in the selection of the case for safety evaluation. If circumstances permit, a discontinuation inspection should be carried out within 14 days of the decision to discontinue (see 2.7.).

2.6. Evaluation items

The primary endpoint of this clinical trial is the disease control rate of non-irradiated target lesions, in order to explore the efficacy (additive effect) of radiotherapy to immunotherapy with nivolumab, which is already established as third-line chemotherapy in unresectable advanced or recurrent gastric cancer. Secondary endpoints will also include 1) median survival time (MST) and 2) safety (grade and frequency of adverse events), 3) local control rate (disease control rate of irradiated target lesions), and 4) immunological parameters.

(1) Primary endpoint

Disease control rate

The rate of patients with a best overall response of stable disease (SD) or better confirmed by 180 days, starting from the start date of radiotherapy (Day 1). CT (MRI is also acceptable) and whole-body PET-CT will be used to determine overall response such as complete response (CR), partial response (PR), SD, progressive disease (PD) at each imaging time according to the RECIST Guidelines Version 1.1. If imaging is not available, the patient is considered deficient (NE). CR or PR as the best overall response is defined as two or more consecutive CRs or PRs. If the best overall response does not meet the criteria for CR or PR, and the patient has no PD on the 6th dose of nivolumab and Day 120, the best overall response is considered to be SD. For evaluation, target lesions other than those treated with local radiotherapy are considered as target lesions. If the lesions cannot be evaluated based on the above criteria, they will not be included in the analysis for best overall response.

(2) Secondary endpoint

1) Median survival time（MST)

Overall survival is defined as the period from the start date of radiotherapy until the date of death from any cause. In surviving cases, the last date of confirmation of survival is the date of termination (survival confirmed by telephone contact is acceptable, but confirmation of survival should be recorded in the medical record). Untraceable cases are terminated on the last date of confirmed survival before the loss of follow-up. At the end of the study period, all enrolled cases will be confirmed alive.

2) Safety (grade and frequency of adverse events)

The frequency of adverse events occurring from the start date of radiotherapy to the end of study will be tabulated by adverse event name and worst grade according to CTCAE ver.4.0. All adverse events are summarized without regard to causal relationships to the study treatment. A serious adverse event is defined as having occurred if at least one of the following adverse events is observed, and the frequency and proportion of serious adverse events is calculated.

① Death during protocol treatment or within 30 days of last protocol treatment.

② Death with undeniable causal relationship to protocol treatment after 31 days of last protocol treatment.

③ Grade 4 infection with neutropenia.

④ Grade 4 non-hematological toxicity (excluding adverse events in the blood/bone marrow).

3) Local control rate

The rate of patients with a best overall response of SD or better confirmed by 180 days, starting from the start date of radiotherapy (Day 1). CT (MRI is also acceptable) and whole-body PET-CT will be used to determine overall response such as complete response (CR), partial response (PR), SD, progressive disease (PD) at each imaging time according to the RECIST Guidelines Version 1.1. If imaging is not available, the patient is considered deficient (NE). CR or PR as the best overall response is defined as two or more consecutive CRs or PRs. If the best overall response does not meet the criteria for CR or PR, and the patient has no PD on the 6th dose of nivolumab and Day 120, the best overall response is considered to be SD. For evaluation, target lesions are tumors that are treated with local radiotherapy. If the lesions cannot be evaluated based on the above criteria, they will not be included in the analysis for best overall response.

4) Immunological parameters

Histological examination

- Analysis of the tumor microenvironment by immunohistochemistry.
  * Immunohistochemistry will be performed in cases with available biopsy and/or resected surgical specimens.

Peripheral blood test

- Analysis of peripheral blood lymphocyte subset
- Measurement of cytokines in plasma
- Analysis of regulatory T cell and antigen-specific T cell function
  * According to advance of immunological technique, the contents of immunological parameter analyses, such as antigen-specific T cell function analysis, highly multiplexed flow cytometric analysis, T cell receptor repertoire analysis, and assessment of tumor mutation burden using plasma derived ctDNA, will also be evaluated.
  * These analysis will be performed relating to clinical outcomes, such as two groups divided with MST.

2.7. Observation and examination items

Pre-registration evaluation items (assess following items once within 14 days prior to date of registration)

1) Patient background (subject identification number, date of obtaining written consent, gender, age, date of birth, height, weight, presence of allergies, medical history, presence of complications, concomitant medications, drinking history, smoking history, history of treatment for cancer).
2) General findings (temperature, blood pressure, pulse).
3) General condition: PS (ECOG).

4) Subjective and objective symptoms: fever, nausea, vomiting, diarrhea, decreased appetite, etc. (to be confirmed according to CTCAE v4.0 Japanese translation).
5) Contrast-enhanced CT (or PET-CT) of chest, abdomen and pelvis, and plus contrast-enhanced MRI if necessary.
6) Blood count (peripheral blood): white blood cell count and fractions, red blood cell count, hemoglobin, MCV, MCH, MCHC, Ht, platelets.
7) Blood biochemistry: total protein, albumin, total bilirubin, direct bilirubin, AST, ALT, ALP, γ-GTP, BUN, creatinine, LDH, uric acid, CPK, P, Ca, Na, K, Cl, CRP, blood sugar, rheumatoid factor (RA), antinuclear antibodies (ANA), SP-D, KL-6, thyroid stimulating hormone (TSH), free triiodothyronine (free T3), free thyroxine (free T4), tumor markers (CEA, CA19-9).
8) Blood coagulation (PT, FDP, D-dimer).
9) Infectious diseases: HIV antigens and antibodies, HTLV-1 antibodies (if testing was performed within 3 months prior to enrollment, the data may be used with the subject's consent).
10) Urinalysis: specific gravity, protein, sugar, occult blood, sedimentation (white blood cells, red blood cells).
11) Pregnancy test (only for women of childbearing potential).
12) Immunological parameters.
13) ECG.

Test items and assessment during and after treatment

After completion of radiotherapy

(Assess the following items once within 14 days of the end date. If the test is performed on the day of the first dose of nivolumab, the same test will be used to evaluate them.)

1) General findings (temperature, blood pressure, pulse).
2) General condition: PS (ECOG).
3) Subjective and objective symptoms: fever, nausea, vomiting, diarrhea, decreased appetite, etc. (to be confirmed according to CTCAE v4.0 Japanese translation).
4) Blood count (peripheral blood): white blood cell count and fractions, red blood cell count, hemoglobin, MCV, MCH, MCHC, Ht, platelets.
5) Blood biochemistry: total protein, albumin, total bilirubin, direct bilirubin, AST, ALT, ALP, γ-GTP, BUN, creatinine, LDH, uric acid, CPK, P, Ca, Na, K, Cl, CRP, blood glucose.
6) Presence of adverse events (CTCAE v4.0 Japanese translation).
7) Immunological parameters.

During nivolumab administration

(After the second dose, assess the following items on the day of administration or the day before administration. Assess following items once from 7 days before to 7 days after the imaging examination date.)

1) General findings (temperature, blood pressure, pulse).
2) General condition: PS (ECOG).
3) Subjective and objective symptoms: fever, nausea, vomiting, diarrhea, decreased appetite, etc. (to be confirmed according to CTCAE v4.0 Japanese translation).
4) Blood count (peripheral blood): white blood cell count and fractions, red blood cell count, hemoglobin, MCV, MCH, MCHC, Ht, platelets.
5) Blood biochemistry: total protein, albumin, total bilirubin, direct bilirubin, AST, ALT, ALP, γ-GTP, BUN, creatinine, LDH, uric acid, CPK, P, Ca, Na, K, Cl, CRP, blood sugar.
   (The following items are only available at the third and sixth doses) RA, ANA, SP-D, KL-6, TSH, free T3, free T4, tumor markers (CEA, CA19-9).
6) Contrast-enhanced CT of the chest, abdomen and pelvis (at third and sixth doses). PET-CT is also acceptable. Add MRI if necessary.
7) Presence or absence of PD (diagnosed comprehensively based on imaging and clinical findings).
8) Presence or absence of adverse events (CTCAE v4.0 Japanese translation).
9) Immunological parameters.

During observation

(Assess following items once from 14 days before to 14 days after the 3rd and 6th dose of Nivolumab and Day120.)

1) Contrast-enhanced CT of chest, abdomen and pelvis. PET-CT is also acceptable. Add MRI if necessary.

After completion of the protocol

(Assess following items once from 14 days before to 14 days after the Day180.)

1) General findings (temperature, blood pressure, pulse).
2) General condition: PS (ECOG).
3) Subjective and objective symptoms: fever, nausea, vomiting, diarrhea, decreased appetite, etc. (to be confirmed according to CTCAE v4.0 Japanese translation).
4) Blood count (peripheral blood): white blood cell count and fractions, red blood cell count, hemoglobin, MCV, MCH, MCHC, Ht, platelets.
5) Blood biochemistry: total protein, albumin, total bilirubin, direct bilirubin, AST, ALT, ALP, γ-GTP, BUN, creatinine, LDH, uric acid, CPK, P, Ca, Na, K, Cl, CRP, blood sugar, RA, ANA, SP-D, KL-6, TSH, free T3, free T4, tumor markers (CEA, CA19-9).

6) Contrast-enhanced CT of chest, abdomen and pelvis. PET-CT is also acceptable. Add MRI if necessary.
7) Presence or absence of PD (diagnosed comprehensively based on imaging and clinical findings).
8) Presence or absence of adverse events (CTCAE v4.0 Japanese translation).
9) Immunological parameters.

At the time of cessation of treatment

(Assess following items once within 14 days after the decision to discontinue. Assessment of following items within 14 days prior to the date of cessation of treatment is valid. If the subject is dead, hospitalized in another hospital, or otherwise unable to come to the hospital, these situations should be noted).

1) General findings (temperature, blood pressure, pulse).
2) General condition: PS (ECOG).
3) Subjective and objective symptoms: fever, nausea, vomiting, diarrhea, decreased appetite, etc. (to be confirmed according to CTCAE v4.0 Japanese translation).
4) Blood count (peripheral blood): white blood cell count and fractions, red blood cell count, hemoglobin, MCV, MCH, MCHC, Ht, platelets.
5) Blood biochemistry: total protein, albumin, total bilirubin, direct bilirubin, AST, ALT, ALP, γ-GTP, BUN, creatinine, LDH, uric acid, CPK, P, Ca, Na, K, Cl, CRP, blood sugar, RA, ANA, SP-D, KL-6, TSH, free T3, free T4, tumor markers (CEA, CA19-9).
6) Contrast-enhanced CT of chest, abdomen and pelvis. PET-CT is also acceptable. Add MRI if necessary.
7) Presence or absence of PD (judged comprehensively based on imaging and clinical findings).
8) Presence or absence of adverse events (CTCAE v4.0 Japanese translation).
9) Immunological parameters.

Inspection schedule

Period	At registration	At the end of radiotherapy	First dose of nivolumab	Second dose of nivolumab	Third dose of nivolumab	Fourth dose of nivolumab	Fifth dose of nivolumab	Sixth dose of nivolumab	Day120	Day180	At the time of treatment cessation
Patient background	○										
General findings	○	○	○	○	○	○	○	○		○	○
General condition	○	○	○	○	○	○	○	○		○	○
Subjective and objective symptoms	○	○	○	○	○	○	○	○		○	○
Imaging	○				○			○	○	○	○
ECG	○										
Blood count	○		○	○	○	○	○	○		○	○
Coagulation	○										
Blood biochemistry	○		○	○	○	○	○	○		○	○
Urinalysis	○										
Infection	○										
Pregnancy test (if required))	○										
Immunological parameters①[#1]	○										
Immunological parameters②[#2]	○		○		○		○			○	○

#1 Histological examination

#2 Peripheral blood test

The volume of blood required for immunological parameters is 30 mL each time.

2.8. Expected number of cases

40 cases.

Basis for setting

Based on a previous study (ONO-4538-12 study) (5), the disease control rate is set at 40%. At a one-side significance level of 5% and a power of 80%, a sample size of 39 cases are required to detect an additional 20% improvement expected from the study treatment.

2.9. Case registration

(1) Case registration

Case enrollment should take place within 14 days prior to the start of radiotherapy (Day 1).

The attending doctors must obtain written consent based on the subject's own free will from those who meet the eligible criteria based on the inclusion/exclusion criteria. The attending doctors then prepare a case registration form that includes the subject identification number, the date of consent obtained, pre-registration assessment items, and the results of the confirmation of the inclusion and exclusion criteria.

A case registration form is prepared for each registered case and faxed to the research office. Research office: Department of Gastrointestinal Tract Surgery, Fukushima Medical University Hospital. FAX number: 024-547-1980 (The case registration forms in Fukushima Medical University Hospital can use an alternative method to fax.)

The case registration form received by the research office is forwarded to the Data Center, which issues a receipt to the chief investigators at each institution, informing them of the registration number. The receipt form is sent to the principal investigator by fax.

In carrying out the above, the chief investigators at each institution prepare a list of subjects and maintain a list of subject identification numbers and registration numbers.

(2) Diagnostic imaging at registration

After case registration, the chief investigators send the imaging data to the research office as soon as possible. Before the start of radiotherapy, prior consultation is held with the Department of Radiation therapy, Fukushima Medical University Hospital regarding radiotherapy and diagnostic imaging at registration.

(3) Notification upon discontinuation of the study

If a subject enrolled in this study meets any of the following items, a discontinuation contact form is prepared and faxed to the research office.

Withdrawal of consent.

Termination of the study due to death.

Withdrawal from the study.

2.10. Statistical analysis

(1) Statistical analysis of the evaluation

The composition of the population to be analyzed shall be as follows:

1) All enrolled cases: the population of enrolled patients, excluding duplicate enrollments and incorrect enrollments.
2) All eligible cases: the population excluding ineligible cases (posterior ineligibility, ineligibility at enrollment, offending enrollment) from all enrolled cases.
3) Largest analyzed population (FAS): all eligible cases for whom treatment was administered.

(2) Method of analysis

Primary endpoints

1) Disease control rate

For FAS cases, summarize the responses of non-irradiated target lesions in a contingency table, and calculate the cumulative disease control rate and the Clopper & Pearson two-side 90% confidence interval. The disease control rate was defined as the total number of patients with CR/PR/SD divided by the number of eligible patients.

Secondary endpoint

1) MST

For FAS cases, estimate the MST using the Kaplan-Meier method, along with a two-sided 95% confidence interval using the Brookmeyer-Crowley method.

2) Safety

For all enrolled cases, data regarding AEs and toxicities were tabulated, and the Clopper & Pearson two-sided 95% CI was calculated.

3) Local control rate

For FAS cases, summarize the responses of irradiated target lesions in a contingency table, and calculate the cumulative disease control rate and the Clopper & Pearson two-side 90% confidence interval. The disease control rate was defined as the total number of patients with CR/PR/SD divided by the number of eligible patients.

4) Immunological parameters

Analyzes are performed at Fukushima Medical University Hospital, Kanagawa Cancer Centre,

and by outsourced testing, as appropriate.

2.11. During the study

Registration period: March 1, 2018 to August 31, 2020

Total study period: March 1, 2018 to August 31. 2021

3. Policy for selecting research subjects

3.1. Inclusion criteria

Subjects who meet all of the following criteria are eligible in this study.

1) Patients with unresectable advanced or recurrent gastric cancer with intolerance or progression after standard treatment (primary and secondary chemotherapy).
2) Patients with more than one measurable lesion and with at least one lesion ≥2 cm as defined by the RECIST guidelines edition 1.1 on diagnostic imaging (whole-body contrast-enhanced CT or PET-CT) within 14 days before entry.
3) Age ≥ 20 years.
4) ECOG performance status (PS): 0–2.
5) No contraindication for nivolumab (anti-PD-1 Ab) administration.
6) No contraindication for radiotherapy.
7) The patient's most recent laboratory tests within 14 days prior to enrollment must meet the following criteria. However, if the clinical examination at the time of enrollment is not included within 7 days prior to the first dose of nivolumab, a repeat examination should be performed within 7 days prior to the first dose of the study drug to reconfirm that the most recent clinical examination prior to the first dose of nivolumab meets the criteria below. In all cases, the laboratory values must not have received a G-CSF product or blood transfusion within 14 days prior to the date of testing.

   White blood cell count ≥ 3,000/µL and neutrophil count ≥ 1,500/µL

   Hemoglobin ≥ 9.0 g/dL

   Platelets ≥ 100,000/µL

   Total bilirubin ≤ 2.0 times the institutional standard upper limit (ISUL)

   AST (GOT) and ALT (GPT) ≤ 3.0 times ISUL (in case with liver metastasis, ≤5.0 times ISUL).

   Serum creatinine ≤ 1.5 times ISUL or creatinine clearance (Ccr) estimate ≥ 60 mL/min.

   Ccr is calculated by the Cockroft-Gault formula:

   Male Ccr = {(140 - age) x weight (kg)}/{72 x serum creatinine level (mg/dL)}

   Female Ccr = 0.85x {(140 - age) x weight (kg)}/{72 x serum creatinine level (mg/dL)}
8) Patients expected survival ≥ 3 months.

9) Written informed consent is obtained from the patient prior to study enrollment.

3.2. Exclusion criteria

Subjects who meet the following criteria are not eligible to enroll in this study:

1) Patients with no tumor lesions that can be irradiated.
2) Patients with metachronous and simultaneous overlapping cancers (excluding intraepithelial cancer of the uterine cervix, fully treated basal cell carcinoma of the skin, and malignant tumors that were treated more than 5 years ago and have not recurred).
3) Patients with a history of severe hypersensitivity reactions to other Ab products.
4) Patients taking immunosuppressive drugs or corticosteroids (prednisone or prednisolone equivalent ≥ 15 mg/day).
5) Patients with active autoimmune diseases or a history of recurrent autoimmune diseases (patients with type-1 diabetes, hypothyroid controllable by hormone replacement therapy, and dermatosis without the need for systemic therapy are eligible).
6) Patients with complications or history of interstitial pneumonia or pulmonary fibrosis diagnosed by imaging studies or clinical findings.
7) Patients with presence of severe disease or medical condition such as severe nutritional deficiencies, transient ischemic attack within 180 days prior to enrollment, cerebral vascular attack within 180 days prior to enrollment, thrombus or thromboembolism within 180 days prior to enrollment, congestive heart failure (NYHA class III or IV), unstable angina, myocardial infarction within 12 months, severe arrhythmias requiring medication, conduction abnormalities such as AV block beyond the second degree, uncontrollable hypertension, liver cirrhosis (Child Class B or higher), mental disorders that may interfere with compliance with this study protocol, unstable diabetes, uncontrolled pericardial fluid, uncontrolled ascites, uncontrolled pleural effusions, diseases requiring anticoagulation therapy (excluding antiplatelet therapy including low-dose aspirin), and systemic infection with treatment.
8) Pregnant or lactating female.
9) Fertile female who are unwilling to use contraception.
10) Fertile male who are not willing to use contraception during study drug administration and for 7 months after study completion (if the partners are fertile females).
11) Prohibited previous treatment

   Within 56 days of registration: radioactive drugs (except radiopharmaceuticals for examination or diagnostic purposes).

   Within 28 days of registration: corticosteroids (excluding temporary use and predonine or prednisolone equivalent ≤15 mg/day), immunosuppressant drugs, anti-cancer drugs, adhesive

treatment of pleura or pericardium, surgery with general anesthesia, and unapproved drugs. Within 14 days of registration: surgery with local or superficial anesthesia.

12) Participating in other clinical trials or clinical studies (excludes those without intervention).
13) Patients with a positive HIV antigen/Ab test or HTLV-1 Ab test.
14) Patients who have history of treatment using ONO-4538, anti-PD-1 Ab, anti-PD-L1 Ab, anti-PD-L2 Ab, anti-CD137 Ab, anti-CTLA-4 Ab, or other Ab or drug therapies for T-cell regulation.
15) Patients determined by the investigator to be ineligible for participation in this study.

4. The scientific rationale for the research

This clinical trial is designed to investigate the additive effect of nivolumab and radiotherapy in patients with unresectable advanced or recurrent gastric cancer with intolerance or progression after standard treatment (primary and secondary chemotherapy), by administering the insured radiotherapy immediately before nivolumab treatment, a third-line treatment indicated by insurance. The results of basic and preclinical studies, as well as those of case reports, suggest that the combination of radiotherapy and anti-cancer immunotherapy may have a synergistic anti-tumor effect. However, no clinical trials similar to this study have been reported to date in patients with gastric cancer, and this study is a phase I/II clinical study exploring safety, clinical efficacy, and immunological changes of the combination therapy using radiotherapy and nivolumab in patients with unresectable advanced or recurrent gastric cancer. The number of planned cases is considered to be the minimum required for statistical purposes.

5. The procedure for obtaining informed consent

5.1. Explanation and consent of research subjects

The attending doctors explain the research to the potential research subjects using a consent document approved by a Certificated Clinical Research Review Committee. At that time, it is also necessary to explain that even after consent to participate in research has been provided, consent can be withdrawn at any time without suffering any particular disadvantages, and that any results presented at conferences, papers, etc. prior to the withdrawal of consent cannot be retracted. After receiving explanations, the research subject is given sufficient time to consider about it and is asked to participate in the trial after confirming that they fully understand the content of the trial. If the research subject agrees to participate in the trial, the research subjects sign the consent document of their own free will. The principal investigator will keep the original consent form, and a copy will be handed to the research subject.

If new information is obtained that may affect the research subject's willingness to continue

participation in the research, the principal investigator will promptly revise the consent document based on the information concerned and obtain the approval of the Certificated Clinical Research Review Committee and permission from the head of the implementing medical institution. In addition, the principal investigator and the attending doctors will explain again using the revised consent document, and obtain written consent from the research subjects to continue their participation in the research.

5.2. Procedures for obtaining informed consent from a substitute or other person

Not applicable

5.3. Procedures for obtaining informed assent

Not applicable

6. Handling of Adverse Events

6.1. Response to Adverse Events

If Grade 3 or higher adverse events and other clinically significant adverse events occur in the period from the start date of radiotherapy to the Day180, regardless of whether they are related to the study or not, the attending doctor will immediately take appropriate action against the research subject, including explanation and treatment for it, and record its progress in the medical record and the case report form.

Determination of known/unknown events: Based on the content of the most recent nivolumab drug information, events that can be classified as equivalent in name and severity are considered as known events. Other events are handled as unknown events.

6.2. Response to the occurrence of serious adverse events

If an adverse event as defined in the preceding paragraph is deemed to be serious, it meets one of the following criteria:

① Resulting in Death,
② Life-threatening,
③ Requiring inpatient hospitalization or prolongation of existing hospitalization for treatment,
④ Resulting in permanent or conspicuous disability or dysfunction,
⑤ Causing congenital anomalies in offspring.

However, hospital admissions stipulated in the research protocol, hospital admissions solely for the purpose of carrying out a therapy or examination planned before the research (before consent was obtained) and during the study (e.g. planned surgery or examinations), and hospital admissions other than for the purpose of therapy or examinations associated with an adverse event (e.g. medical examination) are not treated as serious. In addition, evaluations related to progression of the primary disease or efficacy of treatment are not handled with adverse events.

Attending doctor manage serious adverse events in accordance with the ‘Procedures for handling illnesses, etc.’. In addition, attending doctor report serious adverse events to the Adverse Event Information Desk of Ono Pharmaceutical Industries, Ltd. within 72 hours of receiving the information.

7. Criteria for discontinuation and completion

1) In this study, completion of protocol treatment is defined as completion of six doses of nivolumab (anti-PD-1 Ab) followed by 22.5 Gy/5 doses/5 days of radiotherapy. Study completion is also defined as the end of observation at 6 months (Day180) after the start of treatment. Survival will, however, be confirmed at the end of the study period.
2) Discontinuation of treatment in individual cases is in accordance with '2.5. Criteria for changes of dosage/use'.
3) Discontinuation of the trial is decided by the chief investigator at each institution.
4) The principal investigator will monitor opinions and information from the nivolumab manufacturer and distributor, academic information such as papers, and regulatory information from PMDA and other sources, and will always manage the appropriateness of continuing this study.

The criteria for discontinuation of this clinical trial is defined as follows. If any of the following situations occur and the principal investigator, chief investigators, or administrators of each institution decide that this clinical trial should be terminated, the entire study may be discontinued.

1) Unforeseen serious adverse events occur.
2) The intervention is not effective.
3) Serious violation/noncompliance with the Clinical Research Act or study protocol, etc. is identified.
4) Serious risks to research subjects are identified.

If the principal investigator decides to discontinue the research, he will notify it to the chief investigators, the Certificated Clinical Research Review Committees, the administrators of each

institution, and the Minister of Health, Labour and Welfare within 10 days of the decision. Furthermore, attending doctor will contact the research subjects and inform them of the change in the research schedule. However, even after the notification of discontinuation is submitted, attending doctors make disease reports and periodic reports, etc. until this clinical trial is terminated. The time of termination of this clinical trial after discontinuation is when the procedure for the research subjects is completed.

8. Data management methods

8.1. Prepare and filling out a Case Report Form (CRF)

The CRF is prepared by the Data Center in accordance with the procedures, etc., provided by the principal investigator. If the CRF needs to be revised, the Data Center performs the revision in accordance with the stipulated procedures and inform the chief investigators at each institution.

In principle, completion and correction of the CRF is conducted by the attending doctors. If the attending doctors fill out or revise the CRF, they should obtain confirmation from the chief investigators before submitting the CRF. The attending doctors should complete the CRF as soon as possible after the completion of each observation and examination of each subject.

8.2. Sending and storing Case Report Form (CRF)

The chief investigator sends the CRF to the research office by letter-pack, etc., and the research office submits the received CRF to the Data Center. The CRFs submitted to the Data Center are stored at the Data Center in accordance with the procedures.

8.3. Procedures for amending CRF

Amendments should in principle be made by the attending doctors or others in accordance with the procedure manual. Any amendments should be made using double lines, the reason for the amendment, the date and time of amendment, and the person making the amendment should write them outside the column.

When a correction is required in a CRF that has already been submitted to the Data Center, or when the Data Center makes a questionable data enquiry, the correction should be carried out using a separate enquiry form without adding the correction to the original submitted, and a method should be used to ensure that the history of the correction can be obtained. The attending doctors or others submit the enquiry form to the Data Center.

8.4. Identification of source material

The monitor in charge checks that the case data in CRF are accurate, complete, and consistent with the source documents.

Source documents are the original documents, data, records (e.g. admission records, medical records, examination notes, notes, patient diaries and assessment checklists, administration records, ECG record data, imaging data such as echocardiograms and X-rays, and records kept in the medical technology departments involved in the study such as the pharmacy and laboratory departments).

The following items should be directly recorded in the CRF and should be considered as the source documents.

(1) Reasons for previous and concomitant treatment (medicines and non-pharmacological therapies).

(2) Presence/absence of adverse events, names of adverse events, serious/non-serious of adverse events, treatment, causal relationship with the study drug.

(3) Reasons for discontinuation/termination of the trial and observation.

9. Transfer of samples and information to and from other institution

Transfer of documents and information on registered cases with the Kanagawa Cancer Centre. Transfer is conducted in compliance with ‘11. Handling of personal data’.

When sending samples and information overseas (including cases of outsourcing), the principal investigator prepares a contract, confirmation, memorandum, and a "Record of Provision of Samples and Information" with the overseas recipient, which clearly declares that appropriate procedures equivalent to those of Japanese providers handling personal information.

10. Method of storage and disposal of samples and information

10.1. Storage

Research samples and information are stored as follows:

Objectives for storage	Samples obtained from the humans (serum, DNA samples, etc.).<br>Information obtained from humans (copies of case reports, signed consent forms, test results, CT images, etc.).<br>Materials relating to information used for research*.
The person responsible for storage	Chief investigator at each institution.
Method of	Signed consent forms, case registration forms, copies of CRFs, enquiry forms,

storage	etc., should be kept in accordance with the preservation rules of each institution, and the principal investigator is responsible with them.<br>The principal investigator stored samples and information collected at the research office in a lockable locker in the Department of Gastrointestinal Tract Surgery, Fukushima Medical University School of Medicine. Databases and data sets output from the data management system of the Data Center and electronic data analyzed by computer software, etc., are recorded in two copies on CD-ROM and stored in a lockable locker in the research office.	
Retention period	Samples	5 years from the date of completion of the study or publication of the results.<br>(whichever is later)
	Information<br>Material relating to information used for research	5 years from the date of completion of the study or publication of the results. (whichever is later)

*Materials supporting the information used in the research, such as data modification histories, laboratory notebooks, etc.

Immunological parameter samples obtained from blood collection will be frozen and stored in plasma (-80°C) and PBMC (liquid nitrogen) at each institution. All stored samples will be collected at the research office during the study period and analyzed together at a later date within the study period. The chief investigator will transport all specimens in frozen form to the research office. Samples collected at the research office will be stored in freezer (-80°C) and liquid nitrogen tanks in the Department of Radiation Oncology, Fukushima Medical University School of Medicine.

The retention period shall be until the later of the new research plan or the retention period specified in this clinical trial, if new research is conducted as indicated in ‘22’.

10.2. Disposal

The attending doctors or others shall use the following methods when disposing of samples, information, etc. obtained from the humans.

1） Autoclave samples obtained from the humans.

2） Printed materials should be shredded to a state where they cannot be reproduced, such as by cross cutting with a small cutting size, or by a dissolution process, etc., and then discarded. Electronic data should be physically destroyed to an unreadable level, and then properly discarded.

3） Other materials should also be properly discarded after making their contents unreadable.

11. Handling of personal data

11.1. Method of use

All subjects enrolled in this clinical trial is coded by assigning a unique enrollment number, and high standards of confidentiality will be maintained. A table of codes linked to personal information, such as medical record numbers, and corresponding registration numbers are maintained at the participating institutions only.

The names of subjects, which were collected at the research office, are not recorded on data and samples etc.

11.2. Security management responsibility system

The principal investigator minimizes the risk of information leakage by taking the following safety control measures in the use of personal data.

Physical security control measures	Technical security management measures
The collected subject data are stored on a PC physically separated from the network in the room accessible only to the principal investigator and those authorized by the principal investigator at each institution.	For PCs that store subject data, passwords should be determined so that only the principal investigator at each institution and those authorized by the principal investigator can access the PCs.

In the Data Center in the Clinical Research Center at Fukushima Medical University Hospital, a data security management system is maintained in accordance with the criteria based on the procedure manuals, etc.

12. The burdens and foreseeable risks and benefits to the research subjects, the comprehensive assessment of them and measures to minimize such burdens and risks

Nivolumab treatment is applicable to patients with unresectable advanced or recurrent gastric cancer who have failed to standard therapy (primary and secondary chemotherapy) (intolerance or progression: PD), but response rates and prognosis have not been satisfactory. However, at present, there are no reported outcomes superior to nivolumab therapy for such patients. Radiotherapy is commonly used in patients with unresectable advanced or recurrent gastric cancer, for symptomatic treatment such as pain relief from bone metastases and hemostasis of bleeding from tumors, and for

palliative treatment of liver, lung and brain metastases. Radiotherapy is a local treatment and its efficacy in improving prognosis is poor when multiple lesions are present. Although dozens of clinical trials have been conducted on the use of nivolumab in combination with radiotherapy, there are currently no reliable clinical trial reports showing clear efficacy. However, based on basic and translational research reports and case reports, this combination may be expected to activate systemic anti-tumor immunity and enhance the effect of nivolumab and an abscopal effect may be expected to occur in some cases. A possible disadvantage is that the combination of radiotherapy and nivolumab may increase or enhance adverse events. Although serious adverse events, including deaths, have been reported with nivolumab in combination with other therapies, to the best of our knowledge, there are no reports of serious adverse events occurring in combination with radiotherapy. Of course, at present there are no reliable clinical trial reports on the combination of radiotherapy with nivolumab or immune checkpoint inhibitors other than nivolumab, and we need to proceed with this clinical trial carefully.

All treatments used in this clinical trial are covered by health insurance and are carried out as normal insurance treatment, and treatment costs are covered by the health insurance of the research subject and by the research subjects' co-payment. Therefore, the research subjects will not receive any special medical or financial benefits for participating in this clinical trial.

The combination of radiotherapy and nivolumab may increase the respective adverse events or induce unknown adverse events. To minimize the risk or disadvantage of these adverse events, the selection criteria for study subjects, treatment modification criteria, and concomitant therapy are carefully considered. In addition, the attending doctors also medically monitor the adverse events to assess whether they are within the expected range, and take necessary measures in the event of serious or unanticipated adverse events. Furthermore, efforts should be taken to detect these adverse events at an early stage by conducting the tests, etc. defined in '2.7. Observation and examination items'.

13. The status of funding sources for research and the conflicts of interest related to research of the research institution, individual revenues, and attending doctors, etc.

This clinical trial is funded by an academia-initiated contract research agreement between Ono Pharmaceutical Industries, Ltd./Bristol-Myers Squibb K.K. and Fukushima Medical University. Kanagawa Cancer Centre participates in this clinical trial as a collaborating institution. Prof. Kono, the principal investigator of this clinical trial, has received a speaker's fee from Ono Pharmaceutical Co. The funders, Ono Pharmaceutical Industries, Ltd. and Bristol-Myers Squibb K.K., provide information on the study drug, but not be involved in the planning of this clinical trial, progress of this clinical trial, data collection, analysis, interpretation of results, and reporting. For the purpose of obtaining transparency of financial interests to ensure the fairness and reliability of this clinical trial,

its financial interests is reviewed and approved in advance by the Conflict of Interest Committee of each of Fukushima Medical University and Kanagawa Cancer Center.

The analysis of immunological parameters in this clinical trial will be funded by the research grants obtained by the researchers at Fukushima Medical University.

The principal investigator should decide the planning and release of this clinical trial. Each attending doctor shall manage conflicts of interest in accordance with the conflict of interest policies of the respective academic societies, Fukushima Medical University and Kanagawa Cancer Centre, and disclose them appropriately upon request of the academic societies or medical journals in which they plan to present the results of this clinical trial. Conflicts of interest are also strictly managed at Ono Pharmaceutical Industries, Ltd. and Bristol-Myers Squibb Co.

Furthermore, upon the enforcement of the Clinical Research Act, the following procedures are taken for the management of conflicts of interest in this research. Regarding Article 21 of the Enforcement Regulations of the Act and the Management of Conflicts of Interest in Clinical Research under the Clinical Research Act, the principal investigator prepare the Conflict of Interest Management Criteria based on the "Notification of the Director, Research Development Promotion Division, Medical Affairs Bureau, Ministry of Health, Labour and Welfare, March 2, 2008, Medical Affairs Bureau No. 0302-1" and request each research institution to confirm the conflicts of interest of the declarant (each chief investigator, attending doctors, and persons who clearly benefit from conducting this clinical trial) according to the Conflict of Interest Management Criteria. The Conflict of Interest Management Plan based on these information is reviewed by the Certificated Clinical Research Review Committee.

In the event of new involvement of a person who has reported a conflict of interest, the opinions of the Certificated Clinical Research Review Committee shall be promptly obtained if any changes are necessary to the Conflict of Interest Management Plan after the confirmation by the affiliated institution. Even if there is no change in the Conflict of Interest Management Plan, the principal investigator shall confirm the status of conflicts of interest once a year and report it in the periodic report to maintain transparency and fairness of the research.

14. Methods of disclosing information on research

14.1. Registration of the research plan

The principal investigator registers the research plan in public databases (University Hospital Medical Information Network Clinical Trials Registry (UMIN): http://www.umin.ac.jp/ctr/index-j.htm, and ClinicalTrials.gov: https://clinicaltrials.gov /) to register a summary of the study and update the study information as appropriate.

In addition to the above, the principal investigator also submit and release study information

to Japan Registry of Clinical Trials (jRCT) in accordance with the provisions of the Clinical Research Act that came into effect on April 1, 2018.

14.2. Publication of research results

The principal investigator will register the results of the study in a public database immediately after the study is completed. After taking measures to protect the personal information of the research subjects, the researcher will publish the results of the study in medical journals, etc.

In addition to the above, the principal investigator will also prepare and release the primary endpoint report, comprehensive report, and summary report within the timeframe specified in Article 24 of the Clinical Research Act, which came into effect on April 1, 2018.

Primary endpoint report

The principal investigator will prepare the primary endpoint report in principle within one year after the end of the period for collecting data on the primary endpoint, and promptly report it to the administrator of the implementing medical institution as well as the Certificated Clinical Research Review Committee, and submit it to the Ministry of Health, Labour and Welfare.

Comprehensive report and summary report

The principal investigator will prepare the comprehensive report and summary report in principle within one year after the end of the period for collecting data on all evaluation items, and promptly report them to the administrator of the implementing medical institution as well as the Certificated Clinical Research Review Committee, and submit them to the Ministry of Health, Labour and Welfare. The comprehensive report should include the following items:

1) Background information on the subjects of this clinical trial (age and gender, etc.)
2) Information on the progress according to the design of this clinical trial (number of subjects, etc.)
3) Summary of the occurrence of diseases, etc.
4) Data analysis and results for the primary and secondary endpoints

The Appendix Form 1 should be used to prepare the summary report. The summary report of this clinical trial registered in jRCT will be accepted as the summary report. The following documents should be submitted with the summary report:

1) Study protocol (last revised version)
2) Informed consent form (last revised version)
3) Statistical analysis plan (if any)

The parts of the study protocol that need to be withheld from release for the protection of personal information and intellectual property rights may be published in filled-out form so that their contents

cannot be identified. Since the date when the summary report is recorded in the jRCT is the date of termination, the principal investigator will submit a Notification of Termination to the Minister of Health, Labour and Welfare (Appendix Form 1: Notification of Termination).

15. Report

Periodic report

Once a year, the principal investigator submits a periodic report (Uniform Form 5) to the Certificated Clinical Research Review Committee on the status of implementation of the research. Thereafter, a periodic report (Appendix Form 3) is submitted to the Minister of Health, Labour and Welfare within one month from the date on which the Certificated Clinical Research Review Committee has expressed its opinion.

Non-conformity reporting

If the principal investigator becomes aware of a condition that does not conform to the study protocol (hereinafter referred to as 'non-conformity'), the principal investigator must immediately report it to the administrator of the implementing medical institution. If a particularly serious one is found, the opinion of the Certificated Clinical Research Review Committee must be promptly obtained (Uniform Form 7).

16. Response to consultation from research subjects and others concerned

Questions concerning this clinical trial is handled by the following person.

Contact address: Kosaku Mimura, Gastrointestinal Tract Surgery, Fukushima Medical University Hospital, 1 Hikarigaoka, Fukushima-city, Fukushima 969-1295, Japan
TEL: 024-547-1220

17. Handling of research in situations where there is an immediate and obvious life-threatening situation for the research subject

Not applicable.

18. In cases involving financial burdens or gratuities to research subjects

Not applicable.

19. In the case of research involving invasive procedures, any compensation for damage to health caused by the research

If any health damage occurs to research subjects as a result of the implementation of this clinical trial, the principal investigator will provide appropriate treatment and other necessary measures to the research subjects. In principle, the research subjects will be responsible for paying his/her own medical expenses in such cases. No special compensation, such as medical expenses, medical allowance, or compensation payments, will be provided in the event of health damage resulting from this clinical trial.

20. In the case of research involving medical treatment beyond normal medical treatment, handling of provision of medical treatment to research subjects after completion of the research

The treatment administered in this clinical trial is within the normal range of insurance coverage. There are no special provisions for treatment after completion of observation, but concomitant treatment with unapproved medications, including investigational medications, will not be administered.

21. Handling of research results (including incidental findings) in relation to research subjects for cases in which the conduct of research may lead to important findings, such as the health of the research subjects, genetic characteristics that can be passed on to their offspring, etc.

Not applicable

22. Possibility that the sample/information will be used for future research not identified at the time when consent is received

If new research using stored samples is to be carried out, a new study protocol must be prepared, and the prescribed research ethics committee review and procedures as required by the research content must be performed. Furthermore, the implementation of such new research will be disclosed on the website, and the opportunity for participants in this clinical trial to refuse participation will be guaranteed.

23. Quality control implementation system and procedures for clinical trials

This clinical trial is carried out in compliance with the requirements of this protocol and the ethical principles of the "Ethical Guidelines for Medical Research Involving Human Subjects" (partially revised on February 28, 2017) and the World Medical Association "Declaration of Helsinki" (Fortaleza

revised October 2013). In addition, we manage and conduct this clinical trial in compliance with the Clinical Research Act and related regulations and notifications that were enforced on April 1, 2018.

23.1. Monitoring

Responsible person for implementation: Tomoko Takahashi
Contact: Clinical Research Center, Fukushima Medical University Hospital
TEL: 024-547-1773

The principal investigator prepares the monitoring protocol. The monitoring responsible person conducts monitoring in accordance with the monitoring protocol. When monitoring is conducted, the monitoring responsible person submits a monitoring report to the principal investigator and chief investigators.

23.2. Data center

Responsible person for data management: Takayuki Idaka
Contact: Clinical Research Center, Fukushima Medical University Hospital
TEL: 024-547-1772

The principal investigator prepares the data management protocol. Responsible person for data management operates the Data Center in accordance with the data management protocol.

23.3. Biostatistics

Biostatistician: Shiro Takahashi, Department of Information Science, Iwate Medical University

The biostatistician provides advice on case design and analysis methods.

23.4. Audits

No audits will be carried out.

24. Research implementation organization

Principal investigator: Koji Kono, Gastrointestinal Tract Surgery, Fukushima Medical University

Hospital.

Chief investigators: Koji Kono, Gastrointestinal Tract Surgery, Fukushima Medical University Hospital.

Takashi Oshima, Gastro-esophageal Division, Gastrointestinal Surgery, Kanagawa Cancer Centre.

Research Office: Kosaku Mimura, Gastrointestinal Tract Surgery, Fukushima Medical University Hospital TEL: 024-547-1220

Akihiro Inano, Clinical Research Centre, Fukushima Medical University Hospital TEL: 024-547-1774.

The duties include managing research budget, all research documents, and serving as a contact for research.

Diagnostic imaging at registration: Yoshiyuki Suzuki, Radiation therapy, Fukushima Medical University Hospital TEL: 024-547-1590.

Analysis of immunological parameters

1) Histological examination: Department of Gastrointestinal Tract Surgery, Fukushima Medical University Hospital.

- Analysis of the tumor microenvironment by immunostaining.

2) Peripheral blood tests: Collected samples are sent to contractors by staff in Department of Gastrointestinal Tract Surgery, Fukushima Medical University Hospital.

- Analysis of peripheral blood lymphocyte subset.
- Measurement of cytokines in plasma.

Yohei Miyagi, Director, Clinical Research Institute, Kanagawa Cancer Centre.

- Analysis of regulatory T cell function.
- Analysis of antigen-specific T cell function.

25. Outsourced research and assay

Contractor: ImmunoSCAPE Pte Ltd.

Office address: 1 Scotts Road #24-10, Singapore, 228208

Laboratory address: 8A Biomedical Grove #04-00 Immunos, Singapore, 138648

Contract: Analysis of immunological parameters using peripheral blood

26. Citations

(1) Gastric Cancer Treatment Guidelines, revised May 2014, 4th edition.

(2) Suzuki Y, Mimura K, Yoshimoto Y, Watanabe M, Ohkubo Y, Izawa S, Murata K, Fujii H, Nakano T, Kono K: Immunogenic tumor cell death induced by chemoradiotherapy in patients with esophageal squamous cell carcinoma. *Cancer Res* 2012, 72:3967-3976.

(3) Sato H, Suzuki Y, Yoshimoto Y, Noda SE, Murata K, Takakusagi Y, Okazaki A, Sekihara T, Nakano T: An abscopal effect in a case of concomitant treatment of locally and peritoneally recurrent gastric cancer using adoptive T-cell immunotherapy and radiotherapy. *Clin Case Rep* 2017, 15;5(4):380-384.

(4) Yoshimoto Y, Suzuki Y, Mimura K, Ando K, Oike T, Sato H, Okonogi N, Maruyama T, Izawa S, Noda SE, Fujii H, Kono K, Nakano T: Radiotherapy-induced anti-tumor immunity contributes to the therapeutic efficacy of irradiation and can be augmented by CTLA-4 blockade in a mouse model. *PLoS One* 2014, 31;9(3):e92572.

(5) Kang YK, Boku N, Satoh T, Ryu MH, Chao Y, Kato K, Chung HC, Chen JS, Muro K, Kang WK, Yeh KH, Yoshikawa T, Oh SC, Bai LY, Tamura T, Lee KW, Hamamoto Y, Kim JG, Chin K, Oh DY, Minashi K, Cho JY, Tsuda M, Chen LT. Nivolumab in patients with advanced gastric or gastro-oesophageal junction cancer refractory to, or intolerant of, at least two previous chemotherapy regimens (ONO-4538-12, ATTRACTION-2): a randomised, double-blind, placebo-controlled, phase 3 trial. *Lancet* 2017, 390(10111):2461-2471.

27. Appendix

- Consent Explanatory Document and Consent Form
- Medication package inserts
- PS Scale (ECOG)
