# Supplementary material for: Phase I/II clinical trial of nivolumab in combination with oligo-fractionated irradiation for unresectable advanced or recurrent gastric cancer
Source: Commun Med (Lond). 2023 Aug 15;3:111. doi: 10.1038/s43856-023-00343-4 (PMC10427681; doi:10.1038/s43856-023-00343-4)
Supplement: Supplementary file 3 — Description of Additional Supplementary Files [file 43856_2023_343_MOESM3_ESM.pdf]

Description of Additional Supplementary Files

File Name: Supplementary Data 1

Description: Individual deidentified participant clinical data.
