# Supplementary material for: Phase I/II clinical trial of nivolumab in combination with oligo-fractionated irradiation for unresectable advanced or recurrent gastric cancer
Source: Commun Med (Lond). 2023 Aug 15;3:111. doi: 10.1038/s43856-023-00343-4 (PMC10427681; doi:10.1038/s43856-023-00343-4)
Supplement: Supplementary file 4 — Reporting Summary [file 43856_2023_343_MOESM4_ESM.pdf]

nature portfolio

Corresponding author(s): Koji Kono

Last updated by author(s): Jul 6, 2023

Reporting Summary

Nature Portfolio wishes to improve the reproducibility of the work that we publish. This form provides structure for consistency and transparency in reporting. For further information on Nature Portfolio policies, see our Editorial Policies and the Editorial Policy Checklist.

Statistics

For all statistical analyses, confirm that the following items are present in the figure legend, table legend, main text, or Methods section.

n/a	Confirmed	
☐	☒	The exact sample size (*n*) for each experimental group/condition, given as a discrete number and unit of measurement
☐	☒	A statement on whether measurements were taken from distinct samples or whether the same sample was measured repeatedly
☐	☒	The statistical test(s) used AND whether they are one- or two-sided<br>*Only common tests should be described solely by name; describe more complex techniques in the Methods section.*
☐	☒	A description of all covariates tested
☒	☐	A description of any assumptions or corrections, such as tests of normality and adjustment for multiple comparisons
☐	☒	A full description of the statistical parameters including central tendency (e.g. means) or other basic estimates (e.g. regression coefficient) AND variation (e.g. standard deviation) or associated estimates of uncertainty (e.g. confidence intervals)
☒	☐	For null hypothesis testing, the test statistic (e.g. *F*, *t*, *r*) with confidence intervals, effect sizes, degrees of freedom and *P* value noted<br>*Give P values as exact values whenever suitable.*
☒	☐	For Bayesian analysis, information on the choice of priors and Markov chain Monte Carlo settings
☒	☐	For hierarchical and complex designs, identification of the appropriate level for tests and full reporting of outcomes
☒	☐	Estimates of effect sizes (e.g. Cohen's *d*, Pearson's *r*), indicating how they were calculated

Our web collection on statistics for biologists contains articles on many of the points above.

Software and code

Policy information about availability of computer code

Data collection	Not applicable.
Data analysis	R software (version 4.0.3.).

For manuscripts utilizing custom algorithms or software that are central to the research but not yet described in published literature, software must be made available to editors and reviewers. We strongly encourage code deposition in a community repository (e.g. GitHub). See the Nature Portfolio guidelines for submitting code & software for further information.

Data

Policy information about availability of data

All manuscripts must include a data availability statement. This statement should provide the following information, where applicable:

- Accession codes, unique identifiers, or web links for publicly available datasets
- A description of any restrictions on data availability
- For clinical datasets or third party data, please ensure that the statement adheres to our policy

ClinicalTrials.gov registration: NCT03453164. All relevant data supporting the findings of this study are available within the Article, Supplementary Information file or Supplementary Data.

Research involving human participants, their data, or biological material

Policy information about studies with human participants or human data. See also policy information about sex, gender (identity/presentation), and sexual orientation and race, ethnicity and racism.

Reporting on sex and gender	For both men and women enrolled in this study, there were no preferential selection requirements based on sex and gender.
Reporting on race, ethnicity, or other socially relevant groupings	There were no preferential selection requirements by race, ethnicity, and other socially relevant groupings.
Population characteristics	Patients enrolled in this study had unresectable advanced or recurrent gastric cancer that was intolerance or had progressed after primary and secondary chemotherapy, with more than one lesion assessable in diagnostic imaging (one lesion must be ≥2cm). 41 patients were enrolled in this study. Most of the patients had previously been heavily treated with chemotherapy and tumor burden was relatively high, since most of the patients had developed more than 5 measurable metastatic lesions with more than 3 organs involved.
Recruitment	To be eligible to participate in this study, patients were required to meet the following criteria: (1) unresectable advance or recurrent GC with intolerance or progression after standard treatment (primary and secondary chemotherapy), (2) more than one measurable lesion defined by the Response Evaluation Criteria in Solid Tumors guideline version 1.1 in diagnostic imaging (whole-body contrast-enhanced CT or PET-CT) within 14 days before entry, with at least one lesion ≥2 cm, (3) age: 20≤, (4) eastern cooperative oncology group performance status: 0-2, (5) no contraindication for nivolumab (anti-PD-1 Ab) administration, (6) no contraindication for radiotherapy, (7) the most recent laboratory results within 14 days before study entry fulfill the following: WBC ≥3000/µl, neutrophil ≥1500/µl, hemoglobin ≥9.0g/dl, platelets ≥100,000/µl, total bilirubin ≤2.0 times the institutional standard upper limit (ISUL), AST (GOT) and ALT (GPT) ≤3.0 times ISUL (in case with liver metastasis, ≤5.0 times ISUL), serum creatinine ≤1.5 times ISUL or creatinine clearance ≥ 60 ml/min calculated with cockcroft-Gault equation, (8) expected survival ≥ 3 months.<br>Patients who met the following criteria were not eligible to enroll in this study: (1) no tumor lesions that can be irradiated, (2) metachronous and simultaneous overlapping cancers (excluding intraepithelial cancer of the uterine cervix, fully treated basal cell carcinoma of the skin, and malignant tumors that were treated more than 5 years ago and have not recurred), (3) a history of severe hypersensitivity reactions to other Ab products, (4) taking immunosuppressive drugs or corticosteroids (prednisone or prednisolone equivalent ≥ 15 mg/day), (5) active autoimmune diseases or a history of recurrent autoimmune diseases (patients with type-1 diabetes, hypothyroid controllable by hormone replacement therapy, and dermatosis without the need for systemic therapy are eligible), (6) complications or history of interstitial pneumonia or pulmonary fibrosis diagnosed by imaging studies or clinical findings, (7) presence of severe disease or medical conditions: severe nutritional deficiencies, transient ischemic attack within 180 days prior to enrollment, cerebral vascular attack within 180 days prior to enrollment, thrombus or thromboembolism within 180 days prior to enrollment, congestive heart failure (NYHA class III or IV), unstable angina, myocardial infarction within 12 months, severe arrhythmias requiring medication, conduction abnormalities such as AV block beyond the second degree, uncontrollable hypertension, liver cirrhosis (Child Class B or higher), mental disorders that may interfere with compliance with this study protocol, unstable diabetes, uncontrolled pericardial fluid, uncontrolled ascites, uncontrolled pleural effusions, diseases requiring anticoagulation therapy (excluding antiplatelet therapy including low-dose aspirin), and systemic infection with treatment, (8) pregnant or lactating female, (9) fertile female who are unwilling to use contraception, (10) fertile male who are not willing to use contraception during study drug administration and for 7 months after study completion (if the partners are fertile females), (11) prohibited previous treatment: within 56 days of registration; radioactive drugs (except radiopharmaceuticals for examination or diagnostic purposes), within 28 days of registration; corticosteroids (excluding temporary use and predonine or prednisolone equivalent ≤15 mg/day), immunosuppressant drugs, anti-cancer drugs, adhesive treatment of pleura or pericardium, surgery with general anesthesia, and unapproved drugs, within 14 days of registration; surgery with local or superficial anesthesia, (12) participating in other clinical trials or clinical studies (excludes those without intervention), (13) a positive HIV antigen/Ab test or HTLV-1 Ab test, (14) history of treatment using ONO-4538, anti-PD-1 Ab, anti-PD-L1 Ab, anti-PD-L2 Ab, anti-CD137 Ab, anti-CTLA-4 Ab, or other Ab or drug therapies for T-cell regulation, (15) determined by the investigator to be ineligible for participation in this study.
Ethics oversight	The trial was conducted in accordance with the ethical principles of the 1964 Declaration of Helsinki and its later amendments (ClinicalTrials.gov identifier: NCT03453164, Japan Registry of Clinical Trials identifier: jRCTs021180002, University Hospital Medical Information Network Clinical Trials Registry identifier: UMIN000031508). The trial protocol was approved by the Certified Review Board in Fukushima Medical University School of Medicine (Reference No. 18004) and all patients provided written informed consent before enrolment.

Note that full information on the approval of the study protocol must also be provided in the manuscript.

Field-specific reporting

Please select the one below that is the best fit for your research. If you are not sure, read the appropriate sections before making your selection.

☒ Life sciences ☐ Behavioural & social sciences ☐ Ecological, evolutionary & environmental sciences

For a reference copy of the document with all sections, see nature.com/documents/nr-reporting-summary-flat.pdf

Life sciences study design

All studies must disclose on these points even when the disclosure is negative.

Sample size	Based on a previous study of ATTRACTION-2, the disease control rate was set at 40%. At a one-sided significance level of 5% and a power of 80%, a sample size of 39 patients was needed to detect an additional 20% improvement expected from the study treatment and 41 patients were enrolled in this study.
Data exclusions	One patient whose target lesions did not meet the eligible criteria. This patient was excluded in the efficacy analysis including the disease control rate and median survival time.
Replication	This study was a single-arm, non-randomized phase I/II clinical trial in gastric cancer patients. Replication of data set is not applicable for this study.
Randomization	This study was a single-arm, non-randomized phase I/II clinical trial in gastric cancer patients. Randomization is not applicable for this study.
Blinding	This study was a single-arm, non-randomized phase I/II clinical trial in gastric cancer patients. Blinding is not applicable for this study.

Reporting for specific materials, systems and methods

We require information from authors about some types of materials, experimental systems and methods used in many studies. Here, indicate whether each material, system or method listed is relevant to your study. If you are not sure if a list item applies to your research, read the appropriate section before selecting a response.

Materials & experimental systems

n/a	Involved in the study	
☒	☐	Antibodies
☒	☐	Eukaryotic cell lines
☒	☐	Palaeontology and archaeology
☒	☐	Animals and other organisms
☐	☒	Clinical data
☒	☐	Dual use research of concern
☒	☐	Plants

Methods

n/a	Involved in the study	
☒	☐	ChIP-seq
☒	☐	Flow cytometry
☒	☐	MRI-based neuroimaging

Clinical data

Policy information about clinical studies

All manuscripts should comply with the ICMJE guidelines for publication of clinical research and a completed CONSORT checklist must be included with all submissions.

Clinical trial registration	NCT03453164, jRCTs021180002, UMIN000031508.
Study protocol	Radiotherapy of total 22.5 Gy/5 fractions/5 days was given to the largest or symptomatic lesion and the starting day of radiotherapy was set as day 1. Nivolumab was administered in the period of day 15-22 at a dose of 3 mg/kg or 240 mg/body and continued every 2 weeks to a total of 6 administrations.
Data collection	Tumor responses were evaluated after 3- and 6-times administration of nivolumab, and then on day 120 and 180 or at the end of discontinuation according to the Response Evaluation Criteria in Solid Tumors guideline version 1.1. The disease control rate was defined as the total number of patients with complete response/partial response/stable disease divided by the number of eligible patients. The median survival time was defined as the time from the start date of radiotherapy until the date of death from any cause. Toxicities were graded based on the Common Terminology Criteria for Adverse Events version 4.0.
Outcomes	The primary endpoint for this trial was the disease control rate of non-irradiated target lesions and the secondary endpoints were the median survival time, safety (grading and frequency of adverse events), disease control rate of irradiated lesions, and immunological monitoring.
